# A Multidesign Study of 4CMenB Vaccine Effectiveness and Impact on Meningococcal Carriage and Gonorrhea in Northern Territory Adolescents and Young People

**DOI:** 10.1093/ofid/ofag409

**Published:** 2026-08-04

**Authors:** Mark McMillan, Bing Wang, Vicki Krause, Belinda Greenwood-Smith, Rosalind Webby, Jerry Chen, Rob Baird, Joanne Gerrell, Kaylene Prince, Lisa Whop, Lynne Giles, Jana Bednarz, John Kaldor, Heather D’Antoine, Michael Binks, Ross Andrews, Peter Richmond, Kristine Macartney, David Whiley, Andrew Lawrence, Helen Petousis-Harris, James Ward, Helen Marshall

**Affiliations:** Robinson Research Institute and Adelaide Medical School, The University of Adelaide, Adelaide, South Australia, Australia; Vaccinology and Immunology Research Trials Unit, Women's and Children's Health Network, Adelaide, South Australia, Australia; Robinson Research Institute and Adelaide Medical School, The University of Adelaide, Adelaide, South Australia, Australia; Vaccinology and Immunology Research Trials Unit, Women's and Children's Health Network, Adelaide, South Australia, Australia; NT Department of Health, Centre for Disease Control, Northern Territory, Australia; NT Department of Health, Centre for Disease Control, Northern Territory, Australia; NT Department of Health, Centre for Disease Control, Northern Territory, Australia; NT Department of Health, Centre for Disease Control, Northern Territory, Australia; Territory Pathology, Royal Darwin Hospital, Tiwi, Northern Territory, Australia; NT Department of Health, Centre for Disease Control, Northern Territory, Australia; NT Department of Health, Centre for Disease Control, Northern Territory, Australia; National Centre for Aboriginal and Torres Strait Islander Wellbeing Research, Australian National University, Canberra, Australian Capital Territory, Australia; School of Public Health, The University of Adelaide, Adelaide, South Australia, Australia; School of Public Health, The University of Adelaide, Adelaide, South Australia, Australia; SAHMRI Women and Kids Theme, South Australian Health and Medical Research Institute, Adelaide, South Australia, Australia; TheKirby Institute, University of New South Wales, Sydney, New South Wales, Australia; Menzies School of Health Research, Charles Darwin University, Darwin, Northern Territory, Australia; Menzies School of Health Research, Charles Darwin University, Darwin, Northern Territory, Australia; Menzies School of Health Research, Charles Darwin University, Darwin, Northern Territory, Australia; National Centre for Epidemiology and Population Health, Australian National University, Canberra, Australian Capital Territory, Australia; Wesfarmers Centre of Vaccines and Infectious Diseases, Telethon Kids Institute, Perth, Western Australia, Australia; Discipline of Paediatrics, The University of Western Australia (UWA) Medical School, Perth, Western Australia, Australia; National Centre for Immunisation Research and Surveillance, Faculty of Medicine and Health, The University of Sydney, Westmead, New South Wales, Australia; UQ Centre for Clinical Research, The University of Queensland and Pathology Queensland, Brisbane, Queensland, Australia; Adelaide Medical School, The University of Adelaide, Adelaide, South Australia, Australia; Global Vaccine Data Network and School of Population Health, University of Auckland, Auckland, New Zealand; Poche Centre for Indigenous Health, The University of Queensland, Brisbane, Queensland, Australia; Robinson Research Institute and Adelaide Medical School, The University of Adelaide, Adelaide, South Australia, Australia; Vaccinology and Immunology Research Trials Unit, Women's and Children's Health Network, Adelaide, South Australia, Australia

**Keywords:** 4CMenB vaccine, gonorrhea, meningococcal carriage, vaccine effectiveness, adolescents

## Abstract

**Background:**

Emerging evidence suggests the 4CMenB vaccine provides moderate protection against gonorrhea, likely due to the close genetic relationship between *Neisseria gonorrhoeae* and *Neisseria meningitidis*. 4CMenB has an impact on unencapsulated meningococcal oropharyngeal carriage, where outer membrane proteins are exposed, potentially exerting selective pressure on these strains. We assessed the impact of 4CMenB immunization on gonorrhea disease and oropharyngeal meningococcal carriage among adolescents in the Northern Territory (NT), Australia.

**Methods:**

All adolescents aged 14–19 years in the NT were eligible to receive two doses of 4CMenB between 2021 and 2023. Participants had oropharyngeal swabs collected at baseline and 12 months. Vaccine effectiveness (VE) against gonorrhea was assessed using linked notification and immunization data. A Cox proportional hazards model stratified by sex, age, and geography estimated VE. Mixed-effects logistic regression estimated postvaccination odds of meningococcal carriage.

**Results:**

A total of 30 650 adolescents were included in the VE analysis, with 9.6% receiving 2 doses of 4CMenB, and 42.4% of the population identifying as Aboriginal or Torres Strait Islanders. Gonococcal notifications were reduced in vaccinated adolescents (VE 38.4%, 95% CI: 18.6–53.4). Overall, meningococcal carriage increased from 4.0% to 6.2% (OR 1.68, 95% CI: 1.14–2.48), driven by genogroup B and nongroupable strains. Carriage of disease-associated meningococci remained stable.

**Conclusions:**

4CMenB confers modest protection against gonorrhea in a real-world setting with higher adolescent disease prevalence. These findings are important for women, considering infections are often asymptomatic and have the potential for serious complications. The NT has now introduced a 4CMenB infant and adolescent program.

## Teaser

4CMenB vaccination provided modest protection against gonorrhea in a high-prevalence population but no beneficial impact on oropharyngeal meningococcal carriage. These real-world findings support broader 4CMenB implementation for gonorrhea prevention in populations with elevated risks of gonorrhea.

Invasive meningococcal disease (IMD), caused by the bacterium *Neisseria meningitidis*, results in substantial morbidity and mortality worldwide [1, 2]. Transmission occurs through respiratory droplets from close contacts, resulting in oropharyngeal colonization [3]. Age is one of the most significant factors influencing carriage, which typically peaks between the ages of 18 and 19 years [4]. While colonization is rarely followed by invasive disease, reducing the carriage prevalence of disease-associated meningococcal genogroups would prevent transmission and provide indirect population (herd) protection to other vulnerable and unvaccinated groups [1, 5].

While the 4CMenB vaccine has demonstrated effectiveness against IMD [6, 7], recombinant “meningococcal B” vaccines (Bexsero [4CMenB] and Trumenba), developed using novel protein antigen technologies, do not show the same effects on carriage reduction as polysaccharide-conjugate vaccines [8]. However, in a post hoc analysis of a large cluster randomized controlled trial, there was a 29% reduction in nongroupable meningococcal oropharyngeal carriage in the vaccinated group compared to the control group [8]. The observed reduction likely reflects an effect of 4CMenB on unencapsulated meningococci, in which outer membrane proteins are more exposed, with a population 4CMenB program potentially exerting selective pressure on unencapsulated strains.

Gonorrhea, caused by the bacterium *Neisseria gonorrhoeae*, is a sexually transmissible infection (STI). If left untreated, the infection can lead to pelvic inflammatory disease and tubal scarring, increasing the risk of ectopic pregnancy and ultimately infertility in females, and swelling and scarring in the epididymis or testicles in males [9]. In Australia, notifications of gonorrhea have tripled from 36 to 118 per 100 000 population (2008–2017) [10], with higher rates in the Northern Territory (NT), 2456 per 100 000 (January–June 2024) [11]. In 2023, 10 105 *N. gonorrhoeae* isolates were tested for antimicrobial susceptibility, of which 0.22% showed decreased susceptibility to ceftriaxone and 4.5% showed resistance to azithromycin. The number of extensively drug-resistant (XDR) gonorrhea cases has been rising in Australia, with gonorrhea becoming an untreatable infection [12–14]. Northern Territory recorded its first locally acquired XDR gonorrhea case (resistant to ceftriaxone + azithromycin) in 2025 [13].

Emerging evidence suggests that 4CMenB vaccine offers moderate protection against gonorrhea [7, 15–17]. This cross-protection occurs due to the close genetic relationship between *N. gonorrhoeae* and *N. meningitidis*. The 4CMenB vaccine contains 3 recombinant antigens, namely, neisserial heparin binding antigen (NHBA), factor H binding protein (fHbp), and neisserial adhesin A (NadA). In addition to these antigens, the outer membrane vesicle used in the MeNZB vaccine, which expresses *PorA* serosubtype P1.4, was incorporated to create the 4-component vaccine 4CMenB [18, 19]. Outer membrane vesicle (OMV) proteins, including PorB, RmpM, OpcA, FbpA, and FrpB, are homologous between the 2 bacteria [20]. NHBA is expressed by *N. gonorrhoeae*, which may also be a factor in the cross-protection of the 4CMenB vaccine against gonorrhea. The development of a gonorrhea-specific vaccine has been considered the most likely sustainable solution for epidemic control [21].

Although there are renewed efforts to develop a gonorrhea-specific vaccine, previous candidates that reached clinical trials were ineffective [22]. The most optimistic projection for a licensed gonorrhea vaccine is 10 years [22, 23]. The World Health Organization has designated the prevention of gonorrhea as an urgent priority because of the increasing prevalence and emergence of multiantibiotic resistance [24, 25].

This study aimed to provide 4CMenB immunization to young people aged 14–19 years in the NT and assess the impact of the vaccine on gonorrhea and *N. meningitidis* oropharyngeal carriage.

## METHODS

This study comprised 2 components conducted in parallel among adolescents eligible for 4CMenB vaccination:

1. assessment of vaccine effectiveness against *N. gonorrhoeae* infection using linked Northern Territory population-level notification and immunization data, and
2. longitudinal analysis of *N. meningitidis* oropharyngeal carriage before and after vaccination among a subset of participants who were enrolled in the carriage study and completed questionnaires.

### Patient Consent Statement

The study protocol, recruitment materials, information sheet, and consent forms were approved by the Human Research Ethics Committee of the Northern Territory Department of Health and the Menzies School of Health Research (HREC-2019-3507), Western Australian Aboriginal Health Ethics Committee (HREC996), and Central Australian Human Research Ethics Committee (CA-19-3524).

Participants were recruited from either schools or community-based immunization clinics. For recruitment through schools, parental/guardian written consent was required for participants aged <18 years and verbal assent at the time of the study visits. For those recruited through clinics, parental consent was required for participants under the age of 16, while written consent was obtained from those 16 years and over. Later in the study, participants also had the option to provide electronic consent where appropriate. A waiver of consent was used for linking Australian Immunisation Register data and gonorrhea notifications.

### Study Population

The study was undertaken across the NT in Australia, which has a population of 263 417 (March 2025). The NT encompasses Darwin, an urban city, as well as regional and remote communities in the tropical Top End, and Central Australia, which includes the regional town of Alice Springs and surrounding remote desert communities [26]. The Ngaanyatjarra Lands in Western Australia were also included, as their immunization program is aligned with the NT program. All young people aged 14–19 years (born between 2002 and 2009) were eligible to receive 2 doses of the 4CMenB vaccine between 2021 and 2023, in line with the Australian Technical Advisory Group on Immunisation (ATAGI) recommendations. The estimated eligible population was approximately 17 250 adolescents, of whom around 7000 (41%) were Aboriginal, based on 2016 data [27–29].

Recruitment began on 4 March 2021 and was completed by August 2023, with the dose 2 follow-ups completed by June 2024. During 2021, some communities remained subject to COVID-19–related service restrictions and access to some schools in the NT was limited during the COVID-19 vaccine rollout. Trained nurses visited urban, regional, and remote communities and schools to enroll participants. Following consent, participants completed a brief risk factor questionnaire (Supplementary Data), received 2 doses of 4CMenB, and provided oropharyngeal swabs at baseline (visit 1, the same day as the first vaccine dose) and again approximately 12 months later. A small reimbursement was offered to support the time and travel associated with participation.

### 4CMenB Vaccine Effectiveness Against Gonorrhea

#### Study Design and Data Sources

For the 4CMenB vaccine effectiveness evaluation against gonorrhea, the analysis used time to infection and gonococcal notifications reported in the NT and Ngaanyatjarra Lands between 1 March 2021 and 29 February 2024. Adolescents were not individually enrolled or randomized to vaccinated and unvaccinated groups for this analysis; outcomes were compared according to observed vaccination status in the eligible population using linked notification and immunization data. Gonorrhea cases were included if individuals were aged 15–19 years during the study evaluation (born between 1 March 2001 and 28 February 2009). A rolling cohort design was applied, allowing successive birth cohorts to contribute data as they became age-eligible between 1 March 2021 and 1 March 2024. Notification data from the NT Centre for Disease Control (CDC) were linked with immunization records to determine infection rates among vaccinated and unvaccinated adolescents.

#### Time-to-Infection Analysis (Cohort Study Design)

The study evaluation period began on 1 March 2021 and ended on 29 February 2024.

At-risk person-time commenced at the start of the first study year or, for subsequent study years, at the time the individual reached 15 years of age within that study year. At-risk person-time ended on the date of the study's endpoint (ie, first gonococcal infection) or at the end of follow-up, whichever occurred first.

In the survival analysis, exposure was defined as having received 1 or 2 doses of 4CMenB. The primary study endpoint was to compare the risk of the first gonococcal infection in 4CMenB vaccine recipients versus nonrecipients, as measured by the hazard ratio using a cohort study design. A “first infection” was defined as the first positive gonococcal laboratory test or gonococcal notification occurring after the start of at-risk person-time.

In the primary analysis, Cox proportional hazards models were used to estimate hazard ratios (HRs) with 95% confidence intervals (95% CI), stratified by sex (collected as a binary variable), geographic location (Urban Top End [Greater Darwin] and non-Urban Top End, based on published classification) [30], and age (grouped as a categorical variable: 14–16, 17–19, and 20–22 years). These variables were included as stratification variables in the analysis instead of being treated as covariates, due to violations of proportional hazards assumptions. Robust standard errors were used to account for potential misspecification of the variance structure. Aboriginal and Torres Strait Islander status and socioeconomic status (SES) using SEIFA IRSD categories were excluded due to sparse data in some categories, leading to model convergence problems. Each individual's follow-up time was split at the vaccination date and gonococcal diagnosis date to reflect changes in exposure status. The proportional hazards assumption was examined using the Grambsch-Therneau test. Vaccine effectiveness (VE) was calculated as 1 minus the HR estimated from the model.

#### Risk Factor Analysis

Risk factors associated with gonococcal infections were assessed using known demographic details. Information regarding sexual risk factors was not available. Logistic regression with gonococcal infection status as the dependent variable, adjusted for age, sex, Aboriginal and Torres Strait Islander status, SES, and region, was conducted.

All analyses were conducted using Stata Release 18 (StataCorp LLC) [31].

### *N. meningitidis* Carriage

#### Laboratory Process and Carriage Definitions

Oropharyngeal swabs were screened for *N. meningitidis* using PCR detection of *porA* and *ctrA* genes. Those who had *porA* or *ctrA* detected underwent further molecular analysis to determine the genogroup (A, B, C, E, W, X, or Y), as described previously [8, 32]. “Disease-associated” carriage in this paper is defined as having A, B, C, E, W, X, or Y *N. meningitidis* genogroups detected. “Any” carriage includes every sample that has meningococcal DNA (*porA* or *ctrA*) detected, and “nongroupable” carriage is defined as failure to detect genogroup A, B, C, E, W, X, or Y, in those with *porA* or *ctrA* detected.

Samples were frozen and transferred to South Australia. Once thawed, they were plated on selective agar (NIMM, Micro Neisseria Medium [VCTA]), and isolates were extracted for whole genome sequencing analysis of *N. meningitidis*, as previously described [33]. In a subset of 24 samples that did not grow an isolate, the direct probe-capture enrichment WGS (dWGS) method was used on the sample [34].

#### Risk Factor Assessment

The questionnaire collected information from participants about smoking history, household size, recent antibiotic use, intimate kissing, socializing in pubs and clubs, and alcohol use [32]. Remoteness area and index of relative socioeconomic disadvantage (IRSD) were derived using the participant's postcode [35]. Sexual behavior and other STI-related risk factors were not collected, given that participants were as young as 14 years of age and many were recruited through schools.

#### Statistical Analysis and Power Calculation

Analyses were undertaken according to a prespecified statistical analysis plan. Mixed-effects logistic regression was used to estimate the conditional (subject-specific) odds of carriage at swab 2 relative to swab 1. A random effect (random intercept) was included to account for repeated observations within participants. For the primary outcome, missing values for swab 2 were multiply imputed for N = 1441 participants using the method of fully conditional specification (chained equations) under an assumption of missing at random. The conditional imputation model included the carriage result at the earlier timepoint and additional auxiliary variables associated with missingness in the outcome or the outcome itself and was used to create 100 complete datasets for analysis. Results based on imputed data were combined using Rubin's rules to produce final estimates and confidence intervals. Due to small numbers, odds ratios were not calculated for individual genogroups, except for group B and nongroupable carriage. Risk factor models were based on unimputed data and adjusted for potential confounding according to directed acyclic graphs (Supplementary Data) [29].

## RESULTS

Participants enrolled in the carriage component of the study comprise a subset of the gonorrhea vaccine effectiveness analysis, which uses linked notification and immunization data. Between 4 March 2021 and 28 August 2023, a total of 2694 participants consented through schools and clinics in the NT and Ngaanyatjarra Lands to receive the 4CMenB vaccine.

A total of 30 650 adolescents were included in the analysis of 4CMenB vaccine effectiveness against gonorrhea. The proportion of non-Aboriginal and/or Torres Strait Islander adolescents was 52.5% compared to 42.4% of Aboriginal and Torres Strait Islander participants, with 5.2% missing Aboriginal and Torres Strait Islander status. Half, 50.3%, were from a low SEIFA, and 9.6% were vaccinated with 2 doses of 4CMenB (Table 1). Participants identifying as Aboriginal and/or Torres Strait Islander had lower 2-dose 4CMenB uptake than non-Aboriginal participants (766/12 985 [5.9%] vs 2123/16 077 [13.2%]) and a higher proportion with gonorrhea notifications (1269/12 985 [9.8%] vs 37/16 077 [0.2%]) (Table 1; Supplementary Table 2).

**Table 1.** Demographic Characteristics of Individuals Included in the 4CMenB Vaccine Effectiveness Analysis by Vaccination Status

	Unvaccinated (N = 26 874) N (%)	Partially Vaccinated (N = 820) N (%)	Fully Vaccinated (N = 2956) N (%)	Total (N = 30 650) N (%)
Gonorrhea				
Individual without gonorrhea	25 656 (95.5)	784 (95.6)	2904 (98.2)	29 344 (95.7)
Individual with gonorrhea	1218 (4.5)	36 (4.4)	52 (1.8)	1306 (4.3)
Aboriginal and/or Torres Strait Islander status				
Non-Aboriginal and/or Torres Strait Islander	13 619 (50.7)	335 (40.9)	2123 (71.8)	16 077 (52.5)
Aboriginal and/or Torres Strait Islander	11 744 (43.7)	475 (57.9)	766 (25.9)	12 985 (42.4)
Missing	1511 (5.6)	10 (1.2)	67 (2.3)	1588 (5.2)
Sex				
Male	13 989 (52.1)	426 (52.0)	1412 (47.8)	15 827 (51.6)
Female	12 851 (47.8)	394 (48.1)	1544 (52.2)	14 789 (48.3)
Missing	34 (0.1)	0 (0.0)	0 (0.0)	34 (0.1)
Socioeconomic status (SEIFA)				
Low	13 670 (50.9)	475 (57.9)	1283 (43.4)	15 428 (50.3)
Mid	6692 (24.9)	180 (22.0)	886 (30.0)	7758 (25.3)
High	6493 (24.2)	164 (20.0)	781 (26.4)	7438 (24.3)
Missing	19 (0.1)	1 (0.1)	6 (0.2)	26 (0.1)
Geographic location				
Urban Top End (Greater Darwin)	15 311 (57.0)	397 (48.4)	2020 (68.3)	17 728 (57.8)
Non-Urban Top End	11 554 (43.0)	421 (51.3)	936 (31.7)	12 911 (42.1)
Missing	9 (0.03)	2 (0.2)	0 (0.0)	11 (0.04)

### Vaccine Effectiveness of 4CMenB Against Gonorrhea

#### Time-to-Infection Analysis (Cohort Study Design)

Using a Cox proportional hazards model with robust standard errors, stratified by sex, age group, and geographic location (Urban Top End [Greater Darwin] vs non-Urban Top End), the adjusted hazard ratio (aHR) was 0.62 (95% CI: 0.47–0.81, *P* = .001), VE = 38.4% (95% CI: 18.6%–53.4%). Sex, age group, and geographic location were included in the Cox regression as a stratification variable (Table 2). The proportional hazards assumption was assessed based on Schoenfeld residuals, and no significant violations were detected (global test: *χ*^2^ = 2.83, df = 2, *P* = .24).

**Table 2.** Vaccine Effectiveness Estimates Against Gonorrhea

Outcome	Exposure (Dose)	Adjusted HR (95% CI)	VE (95% CI)
Time to infection^a^			
	Post dose 1	HR 0.73 (0.52–1.03) *P* = .075	26.7% (−3.2 to 48.0)
	Post dose 2	HR 0.62 (0.47–0.81) *P* = .001	38.4% (18.6–53.4)
Time to infection^b^ Male (subgroup analysis)			
	Post dose 1	HR 0.82 (0.48–1.42) *P* = .481	17.7% (−41.5 to 52.2)
	Post dose 2	HR 0.75 (0.49–1.14) *P* = .180	25.0% (−14.2 to 50.7)
Time to infection^b^ Female (subgroup analysis)			
	Post dose 1	HR 0.68 (0.44–1.06) *P* = .091	31.6% (−6.2 to 55.9)
	Post dose 2	HR 0.54 (0.37–0.79) *P* = .001	45.9% (21.4–62.7)
Time to infection^c^ Urban Top End (Greater Darwin) (subgroup analysis)			
	Post dose 1	HR 0.59 (0.20–1.70) *P* = .327	41.3% (−70.3% to 79.7%)
	Post dose 2	HR 0.47 (0.24–0.93) *P* = .029	52.8% (7.2%–75.9%)
Time to infection^c^ Non-Urban Top End (subgroup analysis)			
	Post dose 1	HR 0.76 (0.53–1.08) *P* = .129	24.4% (−8.4% to 47.3%)
	Post dose 2	HR 0.66 (0.48–0.89) *P* = .007	34.3% (10.8%–51.7%)

^a^Adjusted using stratified Cox models with strata defined by sex, age group, and geographic location (Urban Top End [Greater Darwin] vs non-Urban Top End).

^b^Adjusted using stratified Cox models with strata defined by age group and geographic location (Urban Top End [Greater Darwin] vs non-Urban Top End).

^c^Adjusted using stratified Cox models with strata defined by sex and age group.

#### Risk Factors Associated With Gonococcal Infections

Adolescents and young adults who were older, female, Aboriginal and/or Torres Strait Islanders, living in mid and low socioeconomic areas, and residing outside the Urban Top End (Greater Darwin) were more likely to have gonococcal infections (Table 3).

**Table 3.** Risk Factors Associated With Gonococcal Infections

Characteristic	Adjusted Odds Ratio	95% CI	*P* Value
Age in years	1.20	1.17–1.24	<.001
Sex			
Male	Ref		
Female	1.71	1.52–1.93	<.001
Aboriginal and Torres Strait Islander status			
Non-Aboriginal and Torres Strait Islander	Ref		
Aboriginal and/or Torres Strait Islander	30.16	21.45–42.40	<.001
Socioeconomic status (SEIFA)			
High	Ref		
Mid	1.46	1.10–1.91	.017
Low	1.45	1.07–2.00	.009
Local Government Area Region			
Urban Top End (Greater Darwin)	Ref		
Remote Top End	1.47	1.21–1.79	<.001
Urban Central (Alice Springs)	4.23	3.48–5.15	<.001
Remote Central	6.04	4.94–7.38	<.001

#### N. meningitidis Carriage

**Figure 1.**
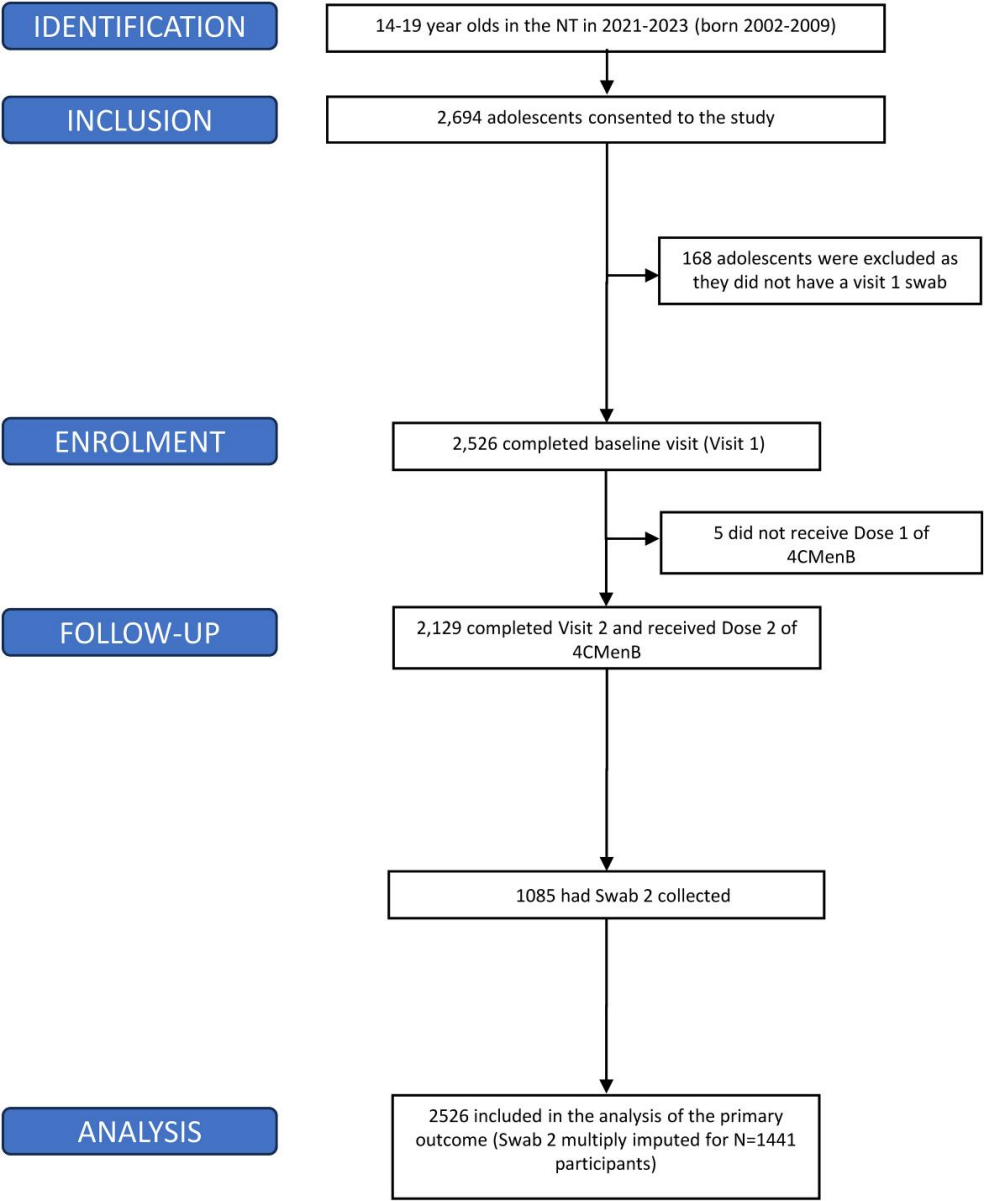
Analysis flow chart.

Of 2694 participants who consented to the carriage study, 168 were excluded because no visit 1 swab was available for analysis (Figure 1). The median time between visit 1 and visit 3 was 370 days (Table 4).

**Table 4.** Baseline Characteristics, Vaccine Uptake, and Follow-Up Time for the Carriage Study

Characteristic	No./Total No. (%)
Gender	
Male	1178/2526 (46.6)
Female	1343/2526 (53.2)
Other	5/2526 (0.2)
Age in years: mean (SD)	16.2 (1.3) (n = 2526)
Aboriginal and Torres Strait Islander status	
Non-Aboriginal and/or Torres Strait Islander	1705/2526 (67.5)
Aboriginal	780/2526 (30.9)
Torres Strait Islander	10/2526 (0.4)
Both Aboriginal and Torres Strait Islander	31/2526 (1.2)
Recruitment region	
Urban Top End (Greater Darwin)	1559/2526 (61.7)
Remote Top End	432/2526 (17.1)
Urban Central (Alice Springs)	250/2526 (9.9)
Remote Central (includes Barkly and NG lands)	285/2526 (11.3)
Vaccination with 4CMenB	
No doses	5/2526 (0.2)
One dose	392/2526 (15.5)
Two doses	2129/2526 (84.3)
Aboriginal and/or Torres Strait Islander vaccination with 4CMenB	
No doses	4/821 (0.5)
One dose	291/821 (35.4)
Two doses	526/821 (64.1)
Non-Aboriginal and Torres Strait Islander vaccination with 4CMenB	
No doses	1/1705 (0.1)
One dose	101/1705 (5.9)
Two doses	1603/1705 (94.0)
Time (days) between vaccine dose 1 and vaccine dose 2^a^: median (IQR)	71.0 (63.0–115.0) (n = 2128)
Time (days) between enrollment visit (visit 1) and visit 3^b^: median (IQR)	370.0 (347.0–457.0) (n = 1083)

Abbreviations: NG lands, Ngaanyatjarra Lands; SD, standard deviation.

^a^Includes all participants with valid dates recorded for both dose 1 and dose 2 of 4CMenB vaccine. Time between dose 1 and dose 2 ranges from 53 to 744 d.

^b^Includes all participants with valid dates recorded for oropharyngeal swab at enrollment visit (swab 1) and oropharyngeal swab at the 12-month visit (swab 2). Time between swab 1 and swab 2 ranges from 78 to 813 d.

#### Baseline Participant Characteristics

The mean age of participants at recruitment was 16.2 (SD 1.3) years of age. The majority of participants were recruited from the most populated urban areas in Darwin and Alice Springs. Aboriginal and/or Torres Strait Islander participants had a lower proportion of completed dose 2 of 4CMenB (64%) compared to non-Aboriginal and Torres Strait Islander participants (94%), as shown in Table 4.

#### Primary Carriage Analysis: Swab 2 vs Swab 1

Based on multiply-imputed data, overall meningococcal carriage increased from 4.0% to 6.2% (OR 1.68 [1.14–2.48], *P* = .01) (Table 5). Carriage of disease-associated meningococci was similar between swab 1 (1.5%) and swab 2 (1.4%). Due to sparse data, odds ratios from mixed-effects models could only be calculated for group B and nongroupable *N. meningitidis* carriage. Nongroupable carriage increased between swab 1 and swab 2.

**Table 5.** *N. meningitidis* Carriage (Swab 2 vs Swab 1)

*N. meningitidis* Genogroup	Swab 1: No./Total No. (%)^a^	Swab 2: No./Total No. (%)^b^	Unadjusted Odds Ratio^c^ (95% CI)	*P* Value
Any *N. meningitidis* detected by porA and/or ctrA PCR (primary outcome)^d^	102 (4.0)	157.6 (6.2)	1.68 (1.14, 2.48)	.01
Any *N. meningitidis* detected by porA PCR	77/2526 (3.0)	39/1085 (3.6)	2.28 (1.04, 4.99)	.04
Any *N. meningitidis* detected by ctrA PCR	79/2526 (3.1)	37/1085 (3.4)	2.88 (1.02, 8.11)	.04
Disease-associated genogroup of *N. meningitidis*^e^	37/2502 (1.5)	15/1076 (1.4)	1.21 (0.52, 2.82)	.66
Group B	23/2502 (0.9)	12/1076 (1.1)	1.65 (0.57, 4.82)	.36
Group C^f^	2/2502 (0.1)	3/1076 (0.3)	-	.25
Group W^f^	3/2502 (0.1)	1/1076 (0.1)	-	1.00
Group X^f^	0/2502 (0.0)	0/1076 (0.0)	-	-
Group Y^f^	3/2502 (0.1)	0/1076 (0.0)	-	1.00
Group E^f^	8/2502 (0.3)	0/1076 (0.0)	-	.50
Nongroupable^g^	37/2502 (1.5)	28/1076 (2.6)	1.78 (1.07, 2.96)	.03

Abbreviations: CI, confidence interval; ctrA PCR, amplification of the capsular transport gene for *N. meningitidis* by polymerase chain reaction; OR, odds ratio; porA PCR, amplification of porA gene from *N. meningitidis* by polymerase chain reaction.

^a^Twenty-four out of 102 (24%) visit 1 swabs testing positive for *N. meningitidis* could not be tested for genogroup.

^b^Nine out of 52 (17%) visit 2 swabs testing positive for *N. meningitidis* could not be tested for genogroup.

^c^Odds ratio from mixed-effects logistic regression describing the conditional (subject-specific) odds of carriage at swab 2 relative to swab 1. A random effect (random intercept) is included to account for repeated observations within participants. Models are based on unimputed data unless otherwise specified.

^d^Defined according to detection of any *N. meningitidis* by porA PCR and/or ctrA PCR. Missing values for swab 2 were multiply imputed for N = 1441 participants. N = 2526 participants are included in the model.

^e^Defined as detection of *N. meningitidis* by porA PCR and/or ctrA PCR plus detection of 1 or more of the following genogroups: A, B, C, E, X, W, Y.

^f^Due to low event rates, mixed-effects logistic regression not performed. *P*-value from McNemar's exact test of paired proportions. There were no instances of group X detected among tested swabs at either timepoint; not analyzed.

^g^Nongroupable carriage defined as detection of *N. meningitidis* by porA PCR and/or ctrA PCR and the absence of detection of any of the following genogroups: A, B, C, E, X, W, Y.

Acquisition of any *N. meningitidis* was defined as negative for any carriage by porA and/or ctrA PCR at enrollment and positive for any carriage by *porA* PCR and/or *ctrA* PCR at 12 months. Using multiply-imputed swab data, it was estimated that 5.6% of participants who had no carriage at baseline would be expected to acquire carriage within the next 12 months (Table 6). Only the primary outcome (any *N. meningitidis* carriage) was imputed, and individual genogroups were not imputed, such that genogroup details are not available for participants whose acquisition status was determined based on multiply imputed 12-month swab data.

**Table 6.** Acquisition of Carriage

	No./Total No. (%) (95% CI)^a^
Acquisition of any *N. meningitidis*^b^	137/2526 (5.4) (3.9, 6.9)
Acquisition of any *N. meningitidis*—carriers at baseline excluded^c^	137/2424 (5.6) (4.1, 7.2)
Acquisition of invasive genogroup(s) of *N. meningitidis*^d^	13/1073 (1.2) (0.6, 2.1)

^a^CIs for multiply-imputed rows were constructed using Rubin’s rules; CI for acquisition of invasive genogroup(s) is exact binomial (Clopper-Pearson) based on unimputed data.

^b^Acquisition of any *N. meningitidis* defined as negative for any carriage by porA and/or ctrA PCR at enrollment and positive for any carriage by porA PCR and/or ctrA PCR at 12 months. Missing 12-month swab data multiply imputed for N = 1441 participants. Acquisition derived using multiply-imputed swab data.

^c^Denominator excludes participants who had carriage detected by porA and/or ctrA PCR at enrollment visit (N = 102 participants.).

^d^Acquisition of disease-causing genogroup(s) of *N. meningitidis* defined as the absence of carriage of disease-causing genogroup(s) at enrollment and the presence of 1 or more disease-causing genogroups at 12 months. Defined only for participants with swab results available at both timepoints (unimputed data). Acquisition of invasive genogroups of *N. meningitidis* is undefined for participants with a positive result for carriage by porA and/or ctrA PCR at either timepoint but who were not genotyped.

#### Association Between Carriage and Selected Participant Characteristics

Analysis of risk factors, adjusted for potential confounders, identified the following characteristics as being associated with carriage: older age, being an Aboriginal and/or Torres Strait Islander young person, living in a remote area, smoking cigarettes in the last month, passive smoking, and 2 or more sharing a bedroom (Supplementary Table 1).

#### Whole Genome Sequencing (WGS)

Potentially due to the harsh environmental conditions in northern Australia and the long distances that many of the samples needed to travel, isolates of *N meningitidis* were only grown from 3 samples, consisting of genogroups B, C, and 1 nongroupable (NG) strain. The NG strain and genogroup C strain were part of clonal complex 41/44, which is usually associated with serogroup B invasive strains; the serogroup C strain has been very rarely isolated from cases of invasive disease, particularly in Scotland (PubMLST database) [36].

Direct WGS was performed on a subset of 24 NT oropharyngeal samples, which were *por*A or *ctrA* PCR positive, but no *N meningitidis* could be cultured. Sixteen of these that passed quality control assessment were submitted to the PubMLST isolate database to confirm typing results (Supplementary Table 3) [34]. There was a lot of strain variation noted in this small set of samples, where meningococcal direct whole genome sequencing was performed. The majority of samples were either nongroupable or the genogroup could not be determined. Clonal complexes usually associated with hypervirulent strains were detected in a number of samples, eg, CC41/44 (ST-32) complex, usually associated with group B disease (1 cnl, 1 NG), ST-41/44 complex (2 genogroup C), and ST-213 (1 genogroup undetermined but often associated with group Y disease). No samples yielded BAST numbers usually associated with hypervirulent strains.

## DISCUSSION

This study showed 4CMenB vaccine offers modest protection against gonorrhea in a high-disease-burden Australian population, with an estimated vaccine effectiveness (VE) of 38.4%. This is the first paper to specifically report on the effectiveness of 4CMenB in Aboriginal and/or Torres Strait Islander people who are at higher risk of both IMD and gonorrhea and could have a significant benefit from a 4CMenB vaccine program. Aboriginal and/or Torres Strait Islander people experience a higher population-level burden of both IMD and gonorrhea. This likely reflects the ongoing impacts of colonization, barriers to culturally safe health care, and higher background exposure in some communities [37], as well as recognized IMD risk factors such as smoking and close-contact living conditions [38].

These findings are consistent with evidence from South Australia, where the 2-dose VE of 4CMenB against gonococcal infections in adolescents in South Australia was 39.1% (95%CI 31.3%–46.0%) in the most recent 5-year analysis [39]. Similar VE estimates for 4CMenB (ranging between 31% and 47%) have been reported internationally, including Australia [7, 17, 39, 40], the United States [41–43], and Italy [44], and also for the New Zealand (MeNZB) vaccine [15, 45], despite differences in study populations, study design and analysis methods [46].

There are, however, other studies that have not shown a significant protective effect, including an open-label randomized trial from France [47] and a Northern California-based observational study [48]. In the French study, recruitment was stopped early following a positive interim analysis result and did not reach the estimated sample size, resulting in a VE of 22% and *P* = .06 in the final analysis. The Californian study, which had a very low 2-dose vaccination rate of 0.2% among all individuals, showed a protective effect in a limited model but was not significant in an expanded model. Given early stopping in the French trial and the extremely low 2-dose uptake (0.2%) in the Californian cohort, these studies may have been underpowered to detect the modest but biologically plausible VE observed in larger, higher-incidence populations.

The duration of this moderate cross-protection is an important consideration. In the 5-year evaluation in South Australia, the vaccine did not demonstrate effectiveness in those who were 5 years or more since vaccination in a subgroup analysis (− 6.3% [95% CI: −44.5% to 21.8%]), suggesting the modest protective effect wanes after 4–5 years [39]. However, the emerging evidence in the field informed recommendations for targeted programs in the United Kingdom and Galicia, Spain, to use 4CMenB for protection against gonococcal infection in groups at higher risk. In the United Kingdom, this program was introduced through specialist sexual health services primarily for gay, bisexual, and other men who have sex with men at increased risk of gonorrhea [49, 50]. In Galicia, Spain, a broader program targeted adults aged 18–65 years at high risk of sexually transmitted infections [51].

Vaccine uptake for this study was lower than anticipated, primarily because the study was conducted during the peak of the COVID-19 pandemic, which posed challenges in accessing communities. Following advice from our reference group, which represented all partners and other stakeholders, we paused study recruitment in remote communities to increase nursing capacity for COVID-19 vaccination and enable our remote research nurses to support the COVID-19 vaccine rollout. Given the low vaccination coverage, the time-to-infection analysis was conducted as the preferred methodology [52].

The risk profile for gonorrhea is different in the NT to that in some other parts of Australia. Men are at higher risk in Sydney and the Australian Capital Territory [53, 54], likely due to high transmission in men who have sex with men. Risk factors associated with gonococcal infections in the NT included being older, female, identifying as an Aboriginal and/or Torres Strait Islander person, living in a lower or medium SEIFA score area, and living in Remote Top End, Urban Central, or Remote Central local government area. A previous study in sexual health clinics located in New South Wales (n = 31), the NT (n = 2), Queensland (n = 7), and Victoria (n = 1) also found that Aboriginal women were at higher risk than non-Aboriginal women of gonococcal infection. First-test positivity was independently associated with greater socioeconomic disadvantage for non-Aboriginal women, but not Aboriginal or Torres Strait Islander women. For Aboriginal women, living in regional and remote areas was also an independent risk factor, but not for non-Aboriginal women [55]. The observed high incidence in Aboriginal women may, in part, be influenced by higher testing rates in this population, as they are more likely to undergo opportunistic screening. However, the associations observed in the risk factor analysis are likely to be influenced by unmeasured behavioral and healthcare-related factors and should be interpreted as proxies for underlying behavioral, healthcare, and structural factors rather than as direct risk factors themselves. We also note that differences by Indigenous status and geography should be interpreted in the context of broader social and structural determinants of health in the Northern Territory.

This longitudinal study found that 4CMenB vaccination did not result in a decrease in overall carriage of any *N. meningitidis*, and carriage increased in vaccinated individuals over the 12 months. This finding is consistent with current evidence that suggests the recombinant 4CMenB vaccine does not reduce pharyngeal carriage of disease-associated meningococci, including group B meningococci [56]. However, unlike the meningococcal carriage study in South Australia, which found that 4CMenB was associated with a reduction in nongroupable carriage [8], it increased in the NT participants (1.5%–2.6%). It is unclear why nongroupable carriage increased in this population compared to South Australia, but the sample size in the NT study was small in comparison to the SA study, and results are more likely to be robust in a large randomized controlled trial. It may also reflect differences in population characteristics, environmental or transmission dynamics, or natural variation in circulating strains. Given the relatively small number of detections, the exclusion of positive swabs that could not be tested for genotype may also have impacted this finding.

WGS on the 3 *N. meningitidis* cultures and direct WGS on 16 positive meningococcal PCR oropharyngeal samples showed heterogeneity in genogroup, *porA*, ST and clonal complexes as well as BASTs. None of the BAST numbers (where one could be determined) has been associated with hypervirulent strains. There were 3 samples (1 culture and 2 oropharyngeal) where the same strain (genogroup C, ST-206, CC41/44, porA 19-27,15 and BAST 11920) was detected. This strain (or a very close relative) has been rarely associated with invasive disease in Scotland in the late 1980s [36]. It should be noted that there has not been a case of serogroup C invasive meningococcal disease notified from the Northern Territory for at least the last 10 years (NNDSS database, Australia) [57]. Use of direct WGS for *N meningitidis* strain characterization on oropharyngeal samples where no isolate of *N meningitidis* is available is helpful, particularly for genogrouping and *por*A typing. Sensitivity is less for MLST, *fe*tA, and BAST [34].

### Limitations

A limitation of this study was that 2-dose vaccine uptake in the NT was lower than anticipated. The cohort design is arguably better suited to studies such as this, with a low number of the cohort vaccinated [52]. Although the cohort design allowed stratification by region in analyses, it could not ensure that comparison groups had similar risk profiles for sexually transmitted infections. Behavioral risk data, prior STI history, and STI testing frequency were not available in the linked dataset, and vaccinated and unvaccinated adolescents differed in baseline demographic risk factors for gonorrhea. Because gonorrhea notifications depend on testing, differences in opportunistic screening, healthcare contact, and intensity of asymptomatic testing across population subgroups may have influenced the likelihood of diagnosis and biased vaccine effectiveness estimates. These unmeasured and imbalanced factors may have introduced residual confounding in either direction. However, the VE estimates in the current study were similar to those reported in other observational studies, whether a cohort or case-control design was used. Information on immunosuppressive conditions or treatments, including HIV status, CD4 count, chemotherapy, corticosteroid treatment, and other immunosuppressive medications, was not available. Accordingly, the potential influence of immunosuppression on infection risk and vaccine effectiveness could not be assessed.

For the carriage component of the study, the target sample size was not achieved, and not all of the *porA*- or *ctrA*-positive swabs were tested for genotype. Individual-level data were not available for adolescents who did not participate in the carriage study, so the representativeness of the carriage cohort relative to the underlying eligible population could not be directly assessed. However, compared with the Northern Territory population overall, the enrolled carriage cohort included a slightly higher proportion of females and a higher proportion of Aboriginal and Torres Strait Islander participants. A further limitation is the 57.0% missing rate for 12-month swab data, for which the primary outcome was multiply imputed under a missing-at-random assumption. The imputation model included baseline carriage status and auxiliary variables associated with missingness; however, the assumption that missingness depends only on observed characteristics cannot be verified, and residual bias in either direction cannot be excluded. These findings should therefore be interpreted cautiously.

Despite the limitations, the results demonstrate that the 4CMenB vaccine provides modest protection against gonorrhea in a real-world setting, in a population with a higher prevalence of gonorrhea and where rates are higher among women than men. This finding is particularly significant given the potential for serious complications in women, particularly very young women, such as pelvic inflammatory disease, tubal scarring, ectopic pregnancy, and infertility, especially as most infections are asymptomatic. As of January 2025, 4CMenB has been available as part of a funded Territory Government program for infants aged 6 weeks to 12 months and year 9 school students in the NT. Continued monitoring of the impact of the 4CMenB program will be conducted with higher vaccine uptake, as part of the National Health and Medical Research Council Centre for Research Excellence in Neisseria Disease Control, which will allow for estimates of vaccine effectiveness against gonorrhea as vaccination coverage increases in a high-burden-disease and priority group.

## Supplementary Material

ofag409_Supplementary_Data

## Supplementary Data

Supplementary materials are available at *Open Forum Infectious Diseases* online. Consisting of data provided by the authors to benefit the reader, the posted materials are not copyedited and are the sole responsibility of the authors, so questions or comments should be addressed to the corresponding author.
