# Supplementary material for: A Multidesign Study of 4CMenB Vaccine Effectiveness and Impact on Meningococcal Carriage and Gonorrhea in Northern Territory Adolescents and Young People
Source: Open Forum Infect Dis. 2026 Aug 4;13(8):ofag409. doi: 10.1093/ofid/ofag409 (PMC13435330; doi:10.1093/ofid/ofag409)
Supplement: Supplementary file 1 [file ofag409_supplementary_data.pdf]

Supplementary Appendix

A multi-design study of 4CMenB vaccine effectiveness and Impact on meningococcal carriage and gonorrhoea in Northern Territory adolescents and young people

Contents

Supplementary Table 1: Univariate analysis of risk factors for *N. meningitidis* carriage by porA PCR and/or ctrA PCR at enrolment 2

Questionnaire 4

Supplementary Table 2: Demographics characteristics of participants included in the 4CMenB vaccine effectiveness analysis against gonorrhoea 5

Supplementary Table 3: Whole genome and direct sequencing 6

DAGs for analyses of risk factors associated with carriage at baseline 7

Supplementary Table 1: Univariate analysis of risk factors for *N. meningitidis* carriage by porA PCR and/or ctrA PCR at enrolment

Characteristic	*N. meningitidis* detected*: No./total no. (%)	Adjusted OR† (95% CI)	P-value
**Age in years: mean (SD)**	16.8 (1.8) [n=102]	1.38 (1.21, 1.59)	<.01
**Gender**			
Male	49/1178 (4.2)	1.00	
Female	53/1343 (3.9)	0.95 (0.64, 1.41)	0.79
Other‡	0/5 (0.0)	-	
**Aboriginal and/or Torres Strait Islander**			
No	21/1705 (1.2)	1.00	
Yes	81/821 (9.9)	8.78 (5.39, 14.29)	<.01
**Currently attending school**			
No	41/315 (13.0)	1.00	
Yes	61/2206 (2.8)	0.74 (0.42, 1.31)	0.31
**Recruitment region**			
Urban Top End (Greater Darwin)	26/1559 (1.7)	1.00	#<.01
Remote Top End	37/432 (8.6)	5.52 (3.30, 9.23)	<.01
Urban Central (Alice Springs)	4/250 (1.6)	0.96 (0.33, 2.77)	0.94
Remote Central (includes Barkly and NG lands)	35/285 (12.3)	8.25 (4.88, 13.95)	<.01
**Cold or sore throat in past 14 days**			
No	88/2076 (4.2)	1.00	
Yes	13/425 (3.1)	0.74 (0.40, 1.35)	0.32
**Antibiotic use in last month**			
None	95/2201 (4.3)	1.00	#0.27
Stopped in last week	1/86 (1.2)	0.25 (0.03, 1.83)	0.17
Stopped more than one week ago	2/123 (1.6)	0.42 (0.10, 1.74)	0.23
Currently taking	3/97 (3.1)	0.74 (0.23, 2.45)	0.63
**Smoked cigarettes in the last month**			
No	57/2161 (2.6)	1.00	
Yes	45/349 (12.9)	2.24 (1.40, 3.57)	<.01
**Smoked e-cigarettes in the last month**			
No	93/2334 (4.0)	1.00	
Yes	8/179 (4.5)	1.11 (0.52, 2.38)	0.78
**Shared cigarette/e-cigarette in the last month**			
No	70/2200 (3.2)	1.00	
Yes	32/313 (10.2)	1.37 (0.83, 2.24)	0.22
**Chewed tobacco in the last month**			
No	98/2492 (3.9)	1.00	
Yes	4/24 (16.7)	2.10 (0.67, 6.53)	0.20
**Cigarette smoker in the house**			
No	27/1593 (1.7)	1.00	#<.01
Yes, inside the house	26/172 (15.1)	4.83 (2.49, 9.36)	<.01
Yes, outside the house	49/751 (6.5)	2.58 (1.52, 4.39)	<.01
**Days out in the last week**			
0	50/1359 (3.7)	1.00	
1 or more	52/1143 (4.5)	1.23 (0.81, 1.86)	0.34
**Drank alcohol in the past month**			
No	76/2014 (3.8)	1.00	
Yes	26/501 (5.2)	1.68 (0.99, 2.83)	0.05
**Number of drinking days in last month**			
None	81/2160 (3.8)	1.00	#0.14
1	12/212 (5.7)	1.91 (0.95, 3.83)	0.07

Characteristic	*N. meningitidis* detected*: No./total no. (%)	Adjusted OR† (95% CI)	P-value
2 or more	9/138 (6.5)	2.15 (0.97, 4.76)	0.06
**People kissed in the last week**			
0	79/1969 (4.0)	1.00	
1 or more	22/528 (4.2)	0.72 (0.43, 1.21)	0.21
**Number of people sleeping in same room at night**			
1	48/1906 (2.5)	1.00	#<.01
2	27/356 (7.6)	2.06 (1.23, 3.43)	0.01
3	11/123 (8.9)	1.75 (0.85, 3.60)	0.13
4 or more	14/121 (11.6)	2.27 (1.16, 4.46)	0.02

Abbreviations: CI, confidence interval; NG Lands, Ngaanyatjarra Lands; OR, Odds Ratio; SD, standard deviation
* Defined according to detection of any *N. meningitidis* by porA PCR and/or ctrA PCR at enrolment (swab 1).
† Odds Ratio from logistic regression describing the odds of carriage of *N. meningitidis* (at enrolment) relative to the reference category. The Odds Ratio describes the total effect of the risk factor on the odds of carriage. Each model is based on unimputed data and adjusted for potentially confounding variables according to Directed Acyclic Graphs constructed for each exposure-outcome relationship.
‡ There were no instances of carriage among those with 'other' gender. N=5 observations excluded from logistic regression model.
# Overall p-value.

B part of it NT _Questionnaire_version 4.0_10_09_2020[1].pdf 1 5/11/2020 11:14:21 AM

B Part of It NT

Questionnaire

**** This information is completely confidential. Please answer the questions truthfully****

Please tick the appropriate boxes or insert the correct number/date e.g ☑ or ☐☐☐☐

Today's Date ☐☐ / ☐☐ / ☐☐☐☐ (DD/MM/YYYY)

What is your home postcode? ☐☐☐☐☐☐☐☐☐☐☐☐

If you live in a remote community, please provide name of the community

....

1. Do you currently attend school? ☐ Yes ☐ Not in school
2. Have you had a cold or sore throat in the past 14 days? ☐ Yes ☐ No
3. In the last month, have you taken antibiotics?

   ☐ No ☐ Yes, but stopped in the last week

   ☐ Yes, taking antibiotics now ☐ Yes, but stopped more than one week ago

4. In the last month, have you smoked cigarettes? ☐ Yes ☐ No
5. In the last month, have you smoked e-cigarettes? ☐ Yes ☐ No
6. In the last month, have you shared a cigarette/e-cigarette that someone else had smoked?

   ☐ Yes ☐ No

7. In the last month, have you chewed tobacco? ☐ Yes ☐ No
8. Does any other person in the house where you sleep most nights smoke cigarettes?

   ☐ Yes, outside the house ☐ Yes, inside the house ☐ No

9. How many days in the last week have you been to a party, pub, bar, nightclub, sporting event, gathering or sorry business (funeral)?

   ☐ 0 ☐ 1 ☐ 2 ☐ 3 ☐ 4 ☐ 5 ☐ 6 ☐ 7 days

10. In the last month, have you drunk alcohol ? ☐ Yes ☐ No
11. If yes, how many days in the last week did you drink alcohol? ☐☐ Number of days
12. How many people have you kissed (kissing with tongues, not just lips or cheeks) in the last week? ☐☐ Number of people
13. Including yourself, how many people sleep in the same room that you sleep in most nights? ☐☐☐ Number of people

Supplementary Table 2: Demographics characteristics of participants included in the 4CMenB vaccine effectiveness analysis against gonorrhoea

	**Individuals without gonorrhoea (N=29,344) N (%)**	**Individuals with gonorrhoea (N=1,306) N (%)**	**Total (N=30,650) N (%)**
**Aboriginal and Torres Strait Islander status**			
Non-Aboriginal and Torres Strait Islander	16,040 (54.7)	37 (2.8)	16,077 (52.5)
Aboriginal and/or Torres Strait Islander	11,716 (39.9)	1,269 (97.2)	12,985 (42.4)
Missing	1,588 (5.4)	0 (0.0)	1,588 (5.2)
**Sex**			
Male	15,301 (52.1)	526 (40.3)	15,827 (51.6)
Female	14,010 (47.7)	779 (59.7)	14,789 (48.3)
Missing	33 (0.1)	1 (0.1)	34 (0.1)
**Socioeconomic status (SEIFA)**			
Low	14,404 (49.1)	1,024 (78.4)	15,428 (50.3)
Mid	7,539 (25.7)	219 (16.8)	7,758 (25.3)
High	7,375 (25.1)	63 (4.8)	7,438 (24.3)
Missing	26 (0.1)	0 (0.0)	26 (0.1)
**Geographic location**			
Urban Top End (Greater Darwin)	17,537 (59.8)	191 (14.6)	17,728 (57.8)
Non-Urban Top End	11,801 (40.2)	1,110 (85.0)	12,911 (42.1)
Missing	6 (0.0)	5 (0.4)	11 (0.0)
**Dose**			
0	25,656 (87.4)	1,218 (93.3)	26,874 (87.7)
1	784 (2.7)	36 (2.8)	820 (2.7)
≥ 2	2,904 (9.9)	52 (4.0)	2,956 (9.6)

Supplementary Table 3: Whole genome and direct sequencing

ID	Visit	Genogroup	MLST	Clonal complex	PorA	fetA	fHbp	NHBA	NadA	BAST
**From isolate**										
1	1	NG	6058	41/44 complex	18-1,34	F1-5	19	2	0	315
2	2	C	206	41/44 complex	19-27,15	F5-2	19	900	0	1920
3	**2**	B	17461	No CC assigned	22,14	F5-2	1031	new	0	4581
**Direct sequencing**										
4	1	Undetermined	Undetermined	Undetermined	22,14	F1-62/F5-5	-	-	-	Undetermined
5	1	NG	Undetermined	Undetermined	12-6,13-13	F1-47;F5-19	685	129	-	Undetermined
6	1	B	Undetermined	Undetermined	22,14	F5-5	45	18	79	1576
7	1	Undetermined	4145	ST-213 complex	22,14	F5-5	45	18	0	224
8	1	Undetermined	Undetermined	Undetermined	17,9	F3-3	13	-	-	Undetermined
9	1	Undetermined	Undetermined	Undetermined	19,15	F5-1	-	-	-	Undetermined
10	1	NG	823	ST-198 complex	18,25-15	F5-5	4	1678	0	4377
11	1	cnl	34	ST-32 complex	19,15	F5-1	1	20	1	3177
12	1	NG	35	ST-35 complex	22-1,14	F3-9	16	21	0	257
13	1	C	206	ST-41/44 complex	19-27,15	F5-2	19	900	0	1920
14	2	Undetermined	Undetermined	Undetermined	18-1,34	F3-3	19	-	0	Undetermined
15	2	NG	175	ST-175 complex	new,2-28	F5-8	22	9	0	Undetermined
16	2	cnl	Undetermined	Undetermined	18-7,43	F4-17	94	-	-	Undetermined
17	2	C	206	ST-41/44 complex	19-27,15	F5-2	19	900	0	1920
18	2	NG	34	ST-32 complex	19,15	F5-1	1	20	1	3177
19	2	NG	Undetermined	Undetermined	18-1,34	F1-5	19	-	-	Undetermined

DAGs for analyses of risk factors associated with carriage at baseline

B Part of it NT

Immunisation for adolescents against serious communicable diseases

STATISTICAL ANALYSIS PLAN - Appendix 1

DAGs for analyses of risk factors associated with carriage at baseline

Gender 2
Age/year level 3
Ethnicity/indigenous status 4
Currently attending school 5
Remoteness area 7
Cold or sore throat in past 14 days 8
Taken antibiotics in the last month 10
Smoked cigarettes in the last month 12
Smoked e-cigarettes in the last month 14
Shared cigarette/e-cigarette in the last month 16
Chewed tobacco in the last month 18
Other person in house smokes cigarettes 20
Days out in last week (party pub/bar/club/sporting event/gathering/funeral) 22
Drunk alcohol in last month/ No. days in last week drunk alcohol 24
No. people kissed in last week 26
Number of people sleeping in the same room 28

Gender

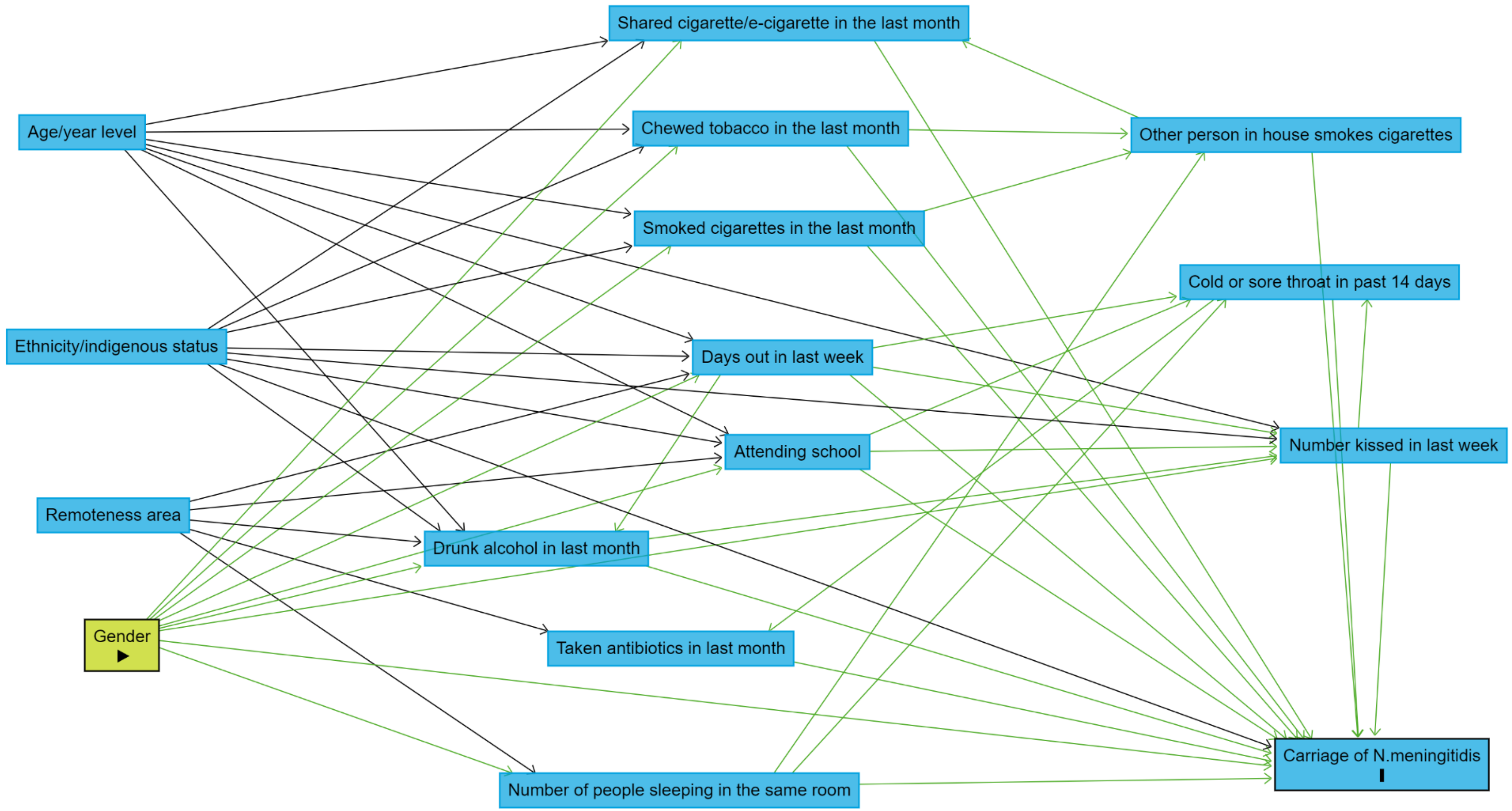

No open biasing paths. No adjustment is necessary to estimate the total effect of Gender on Carriage of N.meningitidis.

Age/year level

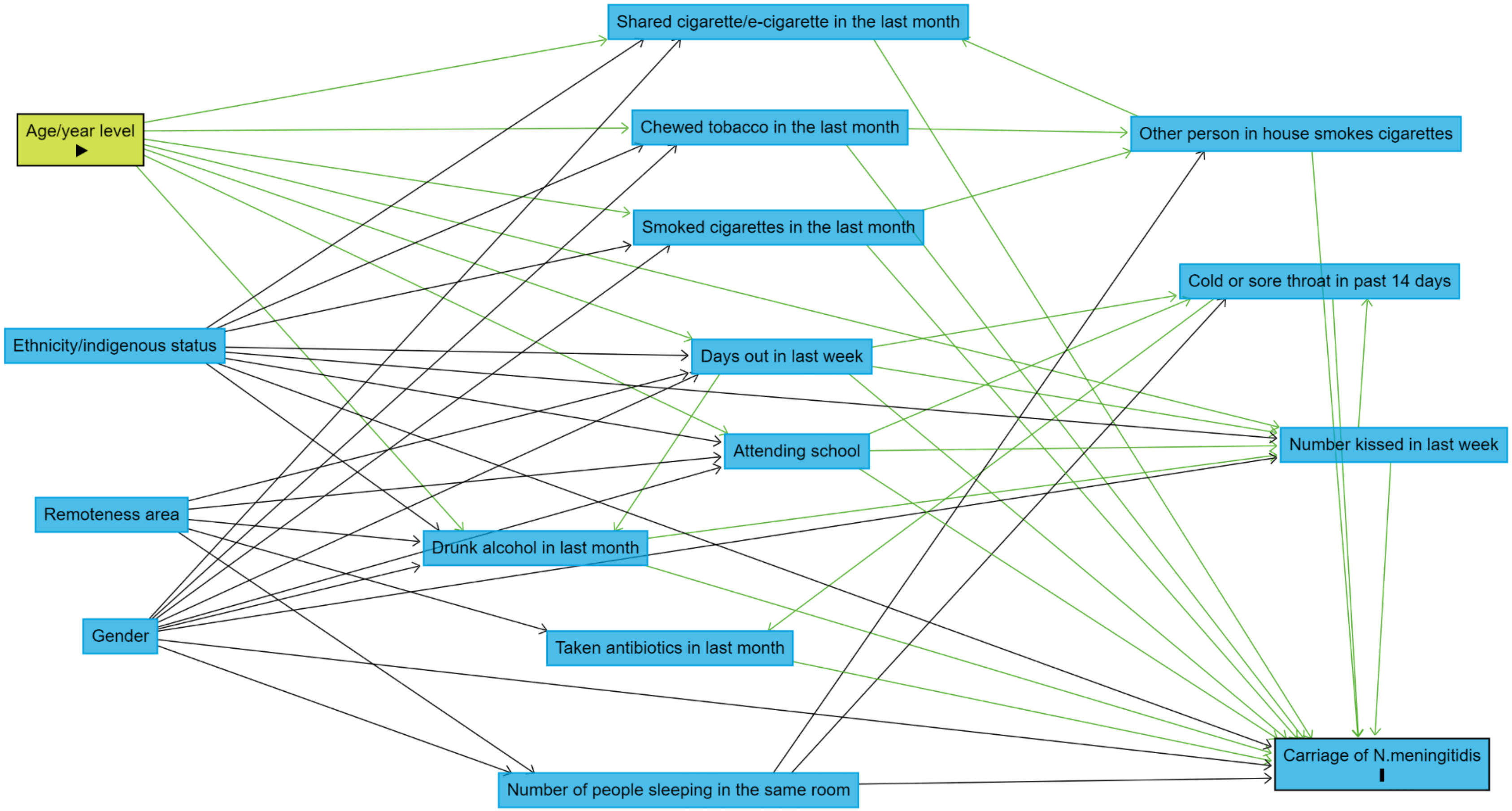

No open biasing paths. No adjustment is necessary to estimate the total effect of age/year level on Carriage of N.meningitidis.

Ethnicity/indigenous status

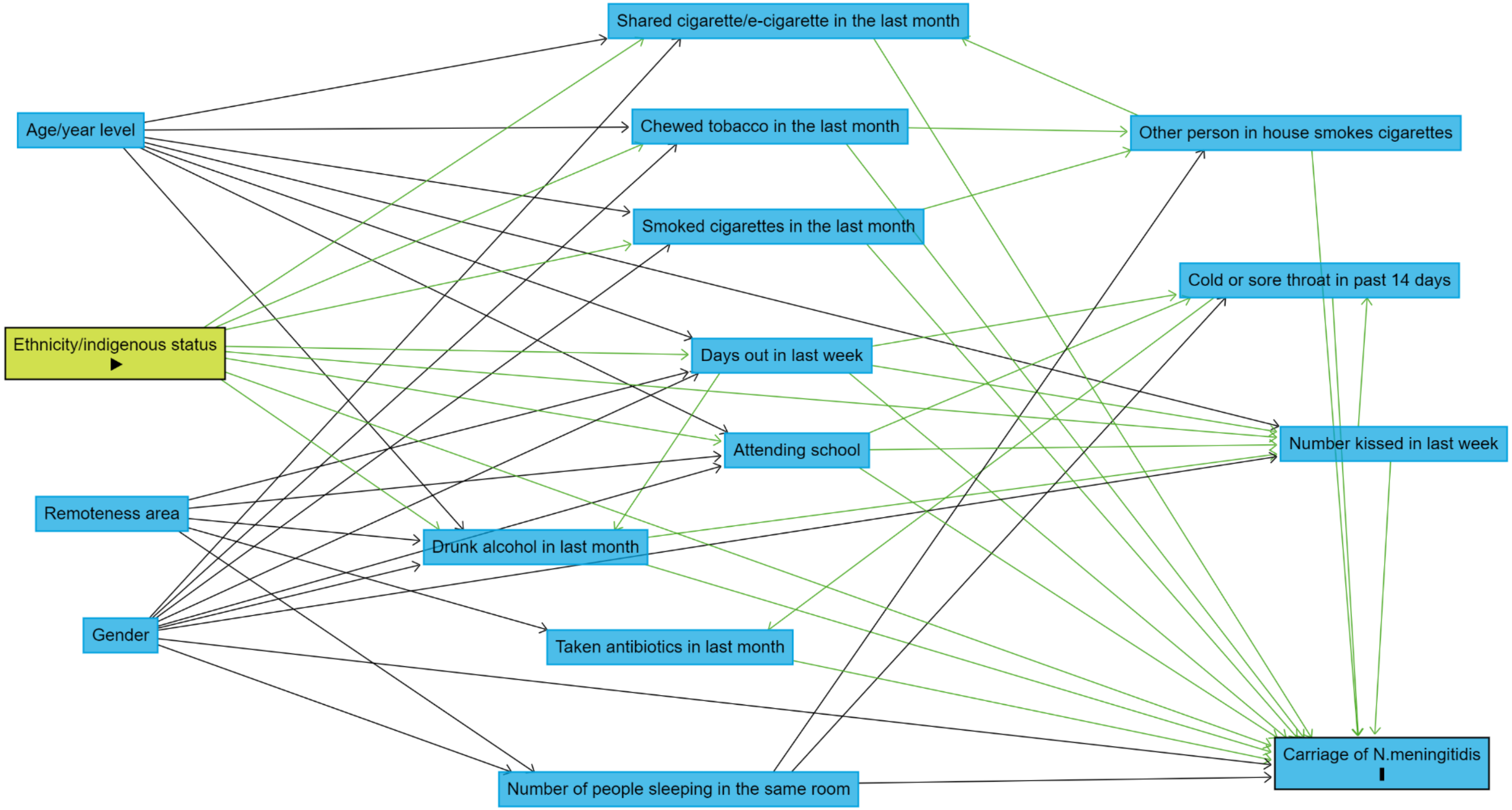

No open biasing paths. No adjustment is necessary to estimate the total effect of ethnicity/indigenous status on Carriage of N.meningitidis.

Currently attending school

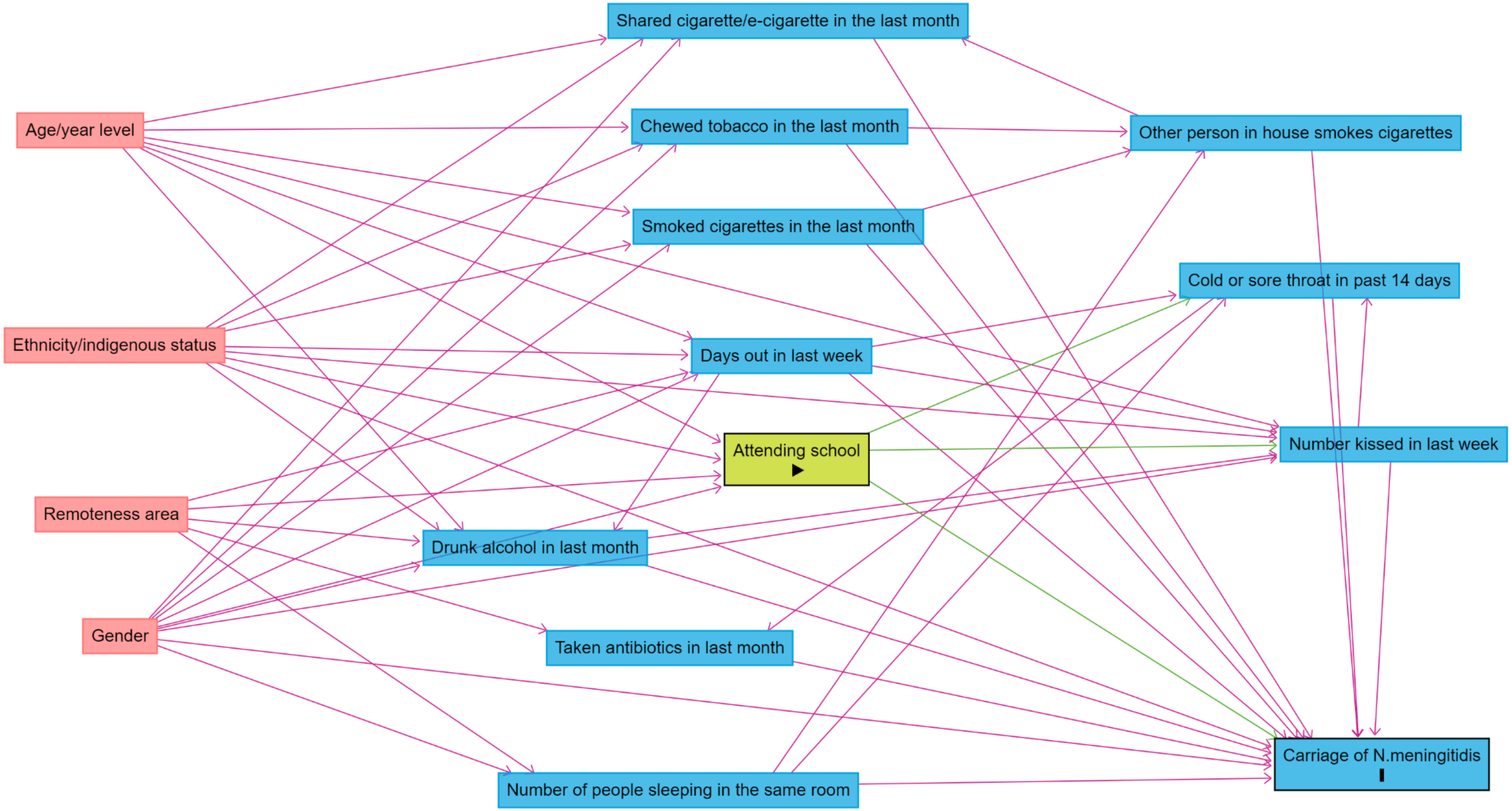

Exposure: Attending school

Outcome: Carriage of N.meningitidis

Biasing paths are open.

Minimal sufficient adjustment sets for estimating the total effect of Attending school on Carriage of N.meningitidis:

- Age/year level, Ethnicity/indigenous status, Gender, Remoteness area

Remoteness area

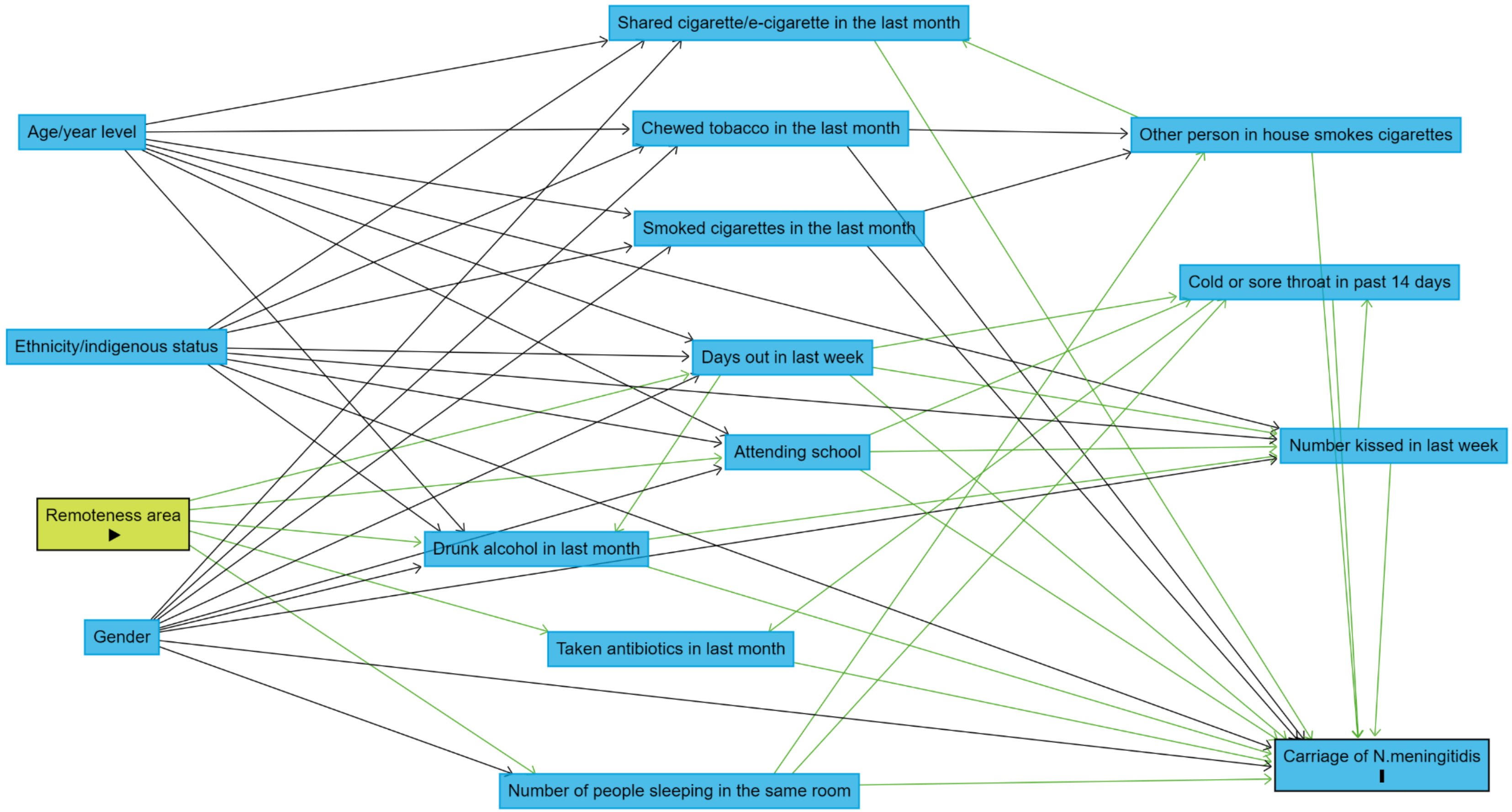

No open biasing paths. No adjustment is necessary to estimate the total effect of Remoteness area on Carriage of N.meningitidis.

Cold or sore throat in past 14 days

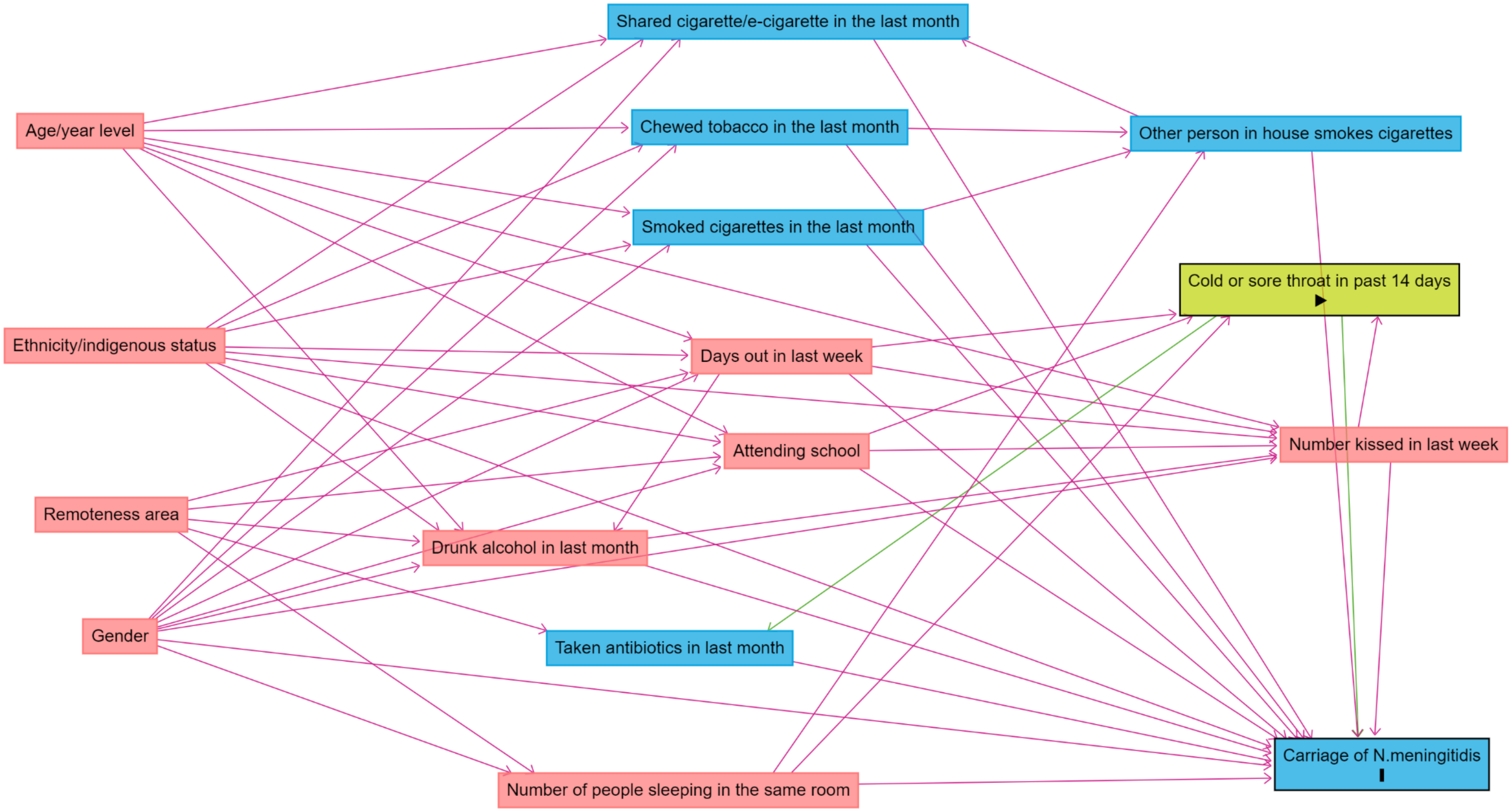

Exposure: Cold or sore throat in past 14 days

Outcome: Carriage of N.meningitidis

Biasing paths are open.

Minimal sufficient adjustment sets for estimating the total effect of Cold or sore throat in past 14 days on Carriage of N.meningitidis:

- Attending school, Days out in last week, Number kissed in last week, Number of people sleeping in the same room

Taken antibiotics in the last month

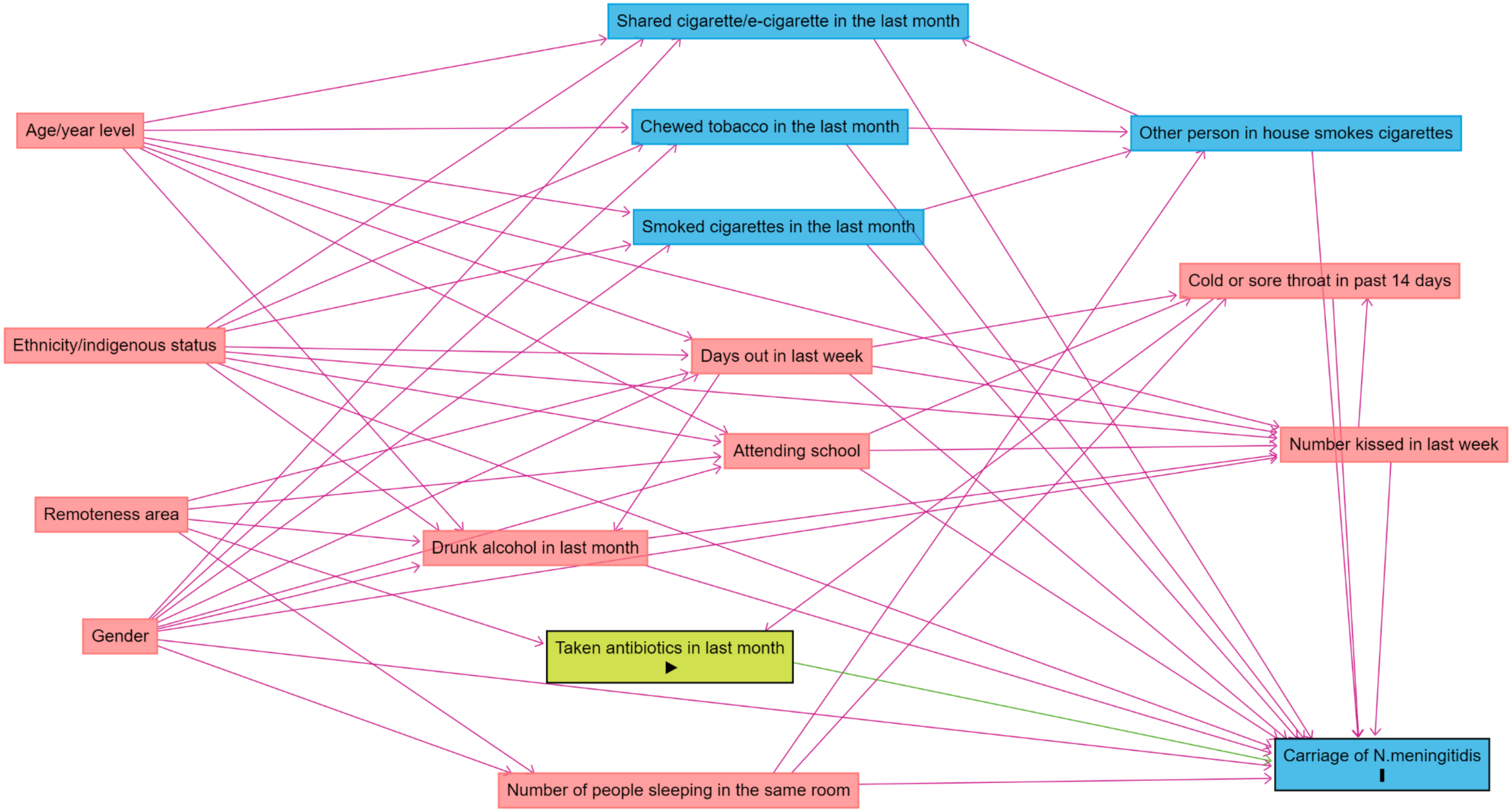

Exposure: Taken antibiotics in last month

Outcome: Carriage of N.meningitidis

Biasing paths are open.

Minimal sufficient adjustment sets for estimating the total effect of Taken antibiotics in last month on Carriage of N.meningitidis:

- Cold or sore throat in past 14 days, Remoteness area

Smoked cigarettes in the last month

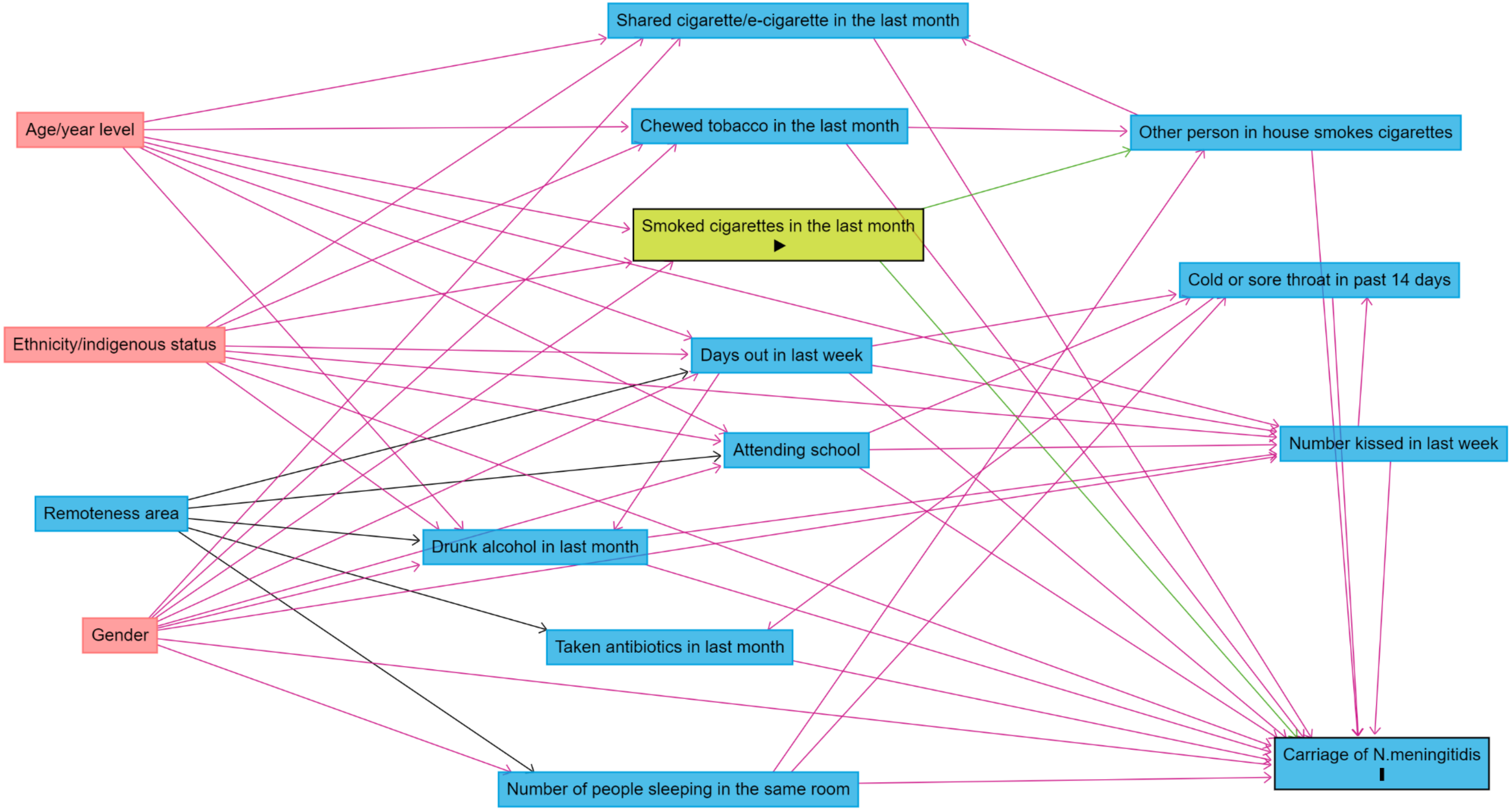

Exposure: Smoked cigarettes in the last month

Outcome: Carriage of N.meningitidis

Biasing paths are open.

Minimal sufficient adjustment sets for estimating the total effect of Smoked cigarettes in the last month on Carriage of N.meningitidis:

- Age/year level, Ethnicity/indigenous status, Gender

Smoked e-cigarettes in the last month

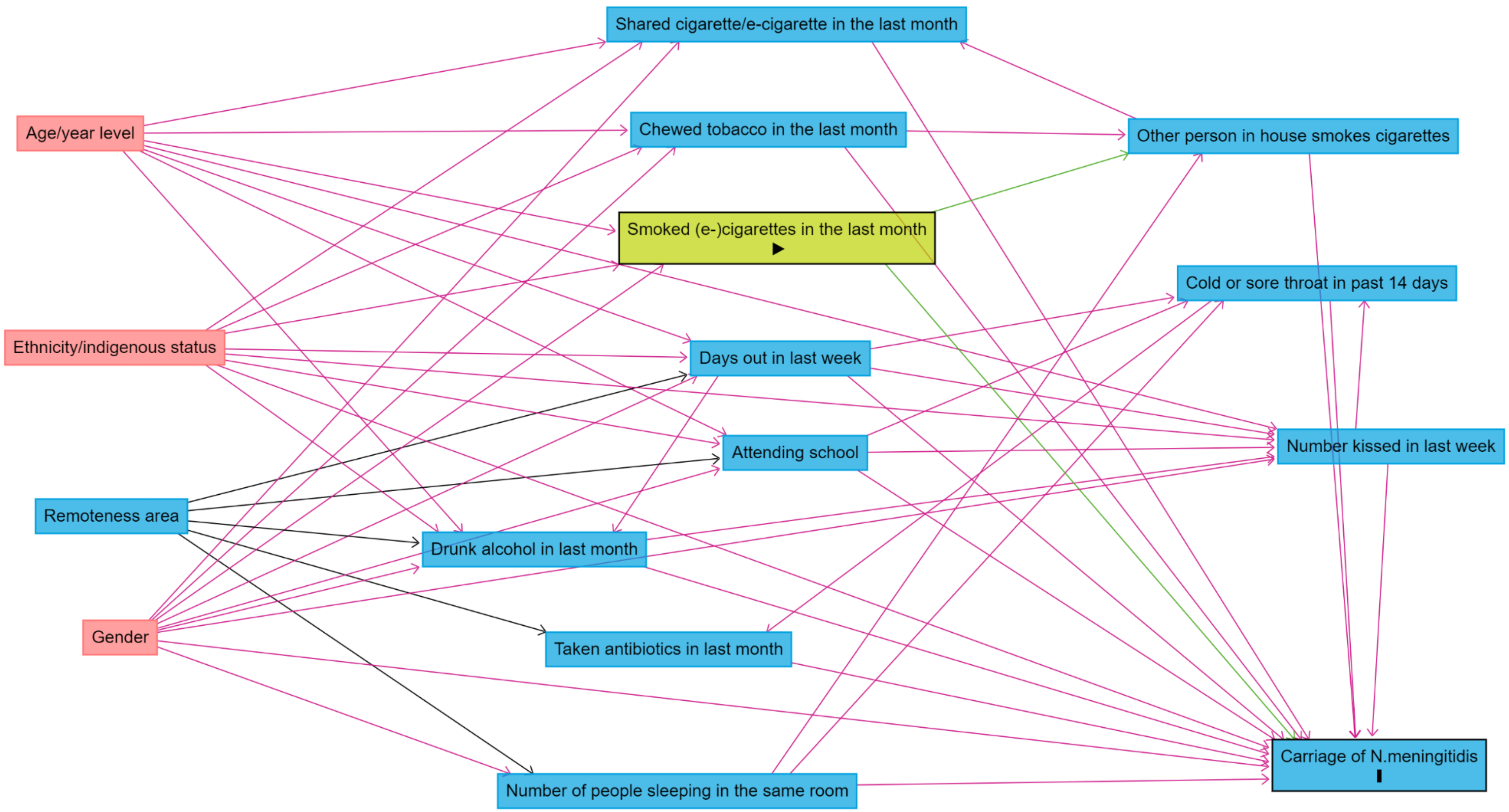

Exposure: Smoked (e-)cigarettes in the last month

Outcome: Carriage of N.meningitidis

Biasing paths are open.

Minimal sufficient adjustment sets for estimating the total effect of Smoked (e-)cigarettes in the last month on Carriage of N.meningitidis:

- Age/year level, Ethnicity/indigenous status, Gender

Shared cigarette/e-cigarette in the last month

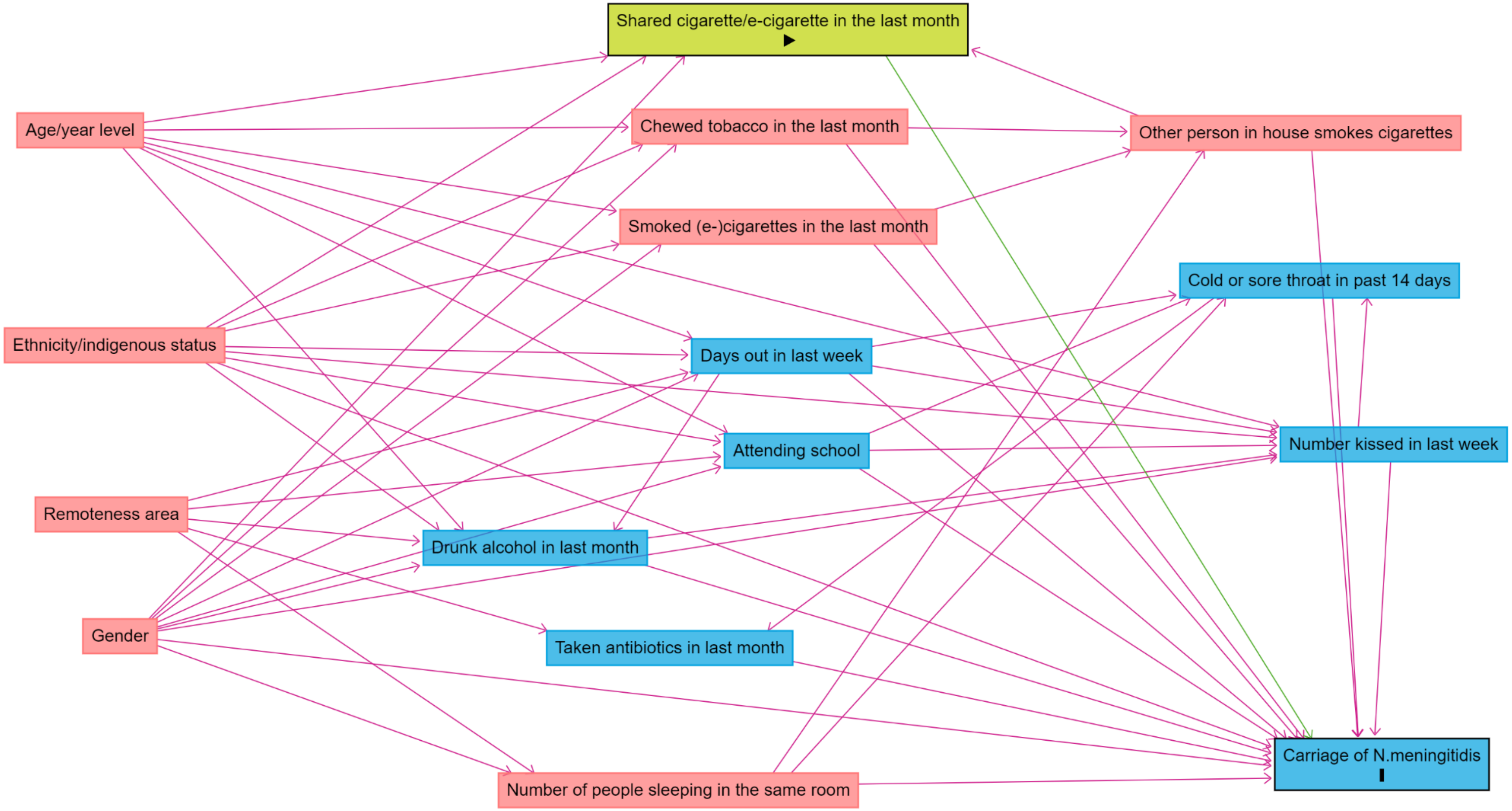

Exposure: Shared cigarette/e-cigarette in the last month
Outcome: Carriage of N.meningitidis
Biasing paths are open.
Minimal sufficient adjustment sets for estimating the total effect of Shared cigarette/e-cigarette in the last month on Carriage of N.meningitidis:

- Age/year level, Ethnicity/indigenous status, Gender, Other person in house smokes cigarettes

Chewed tobacco in the last month

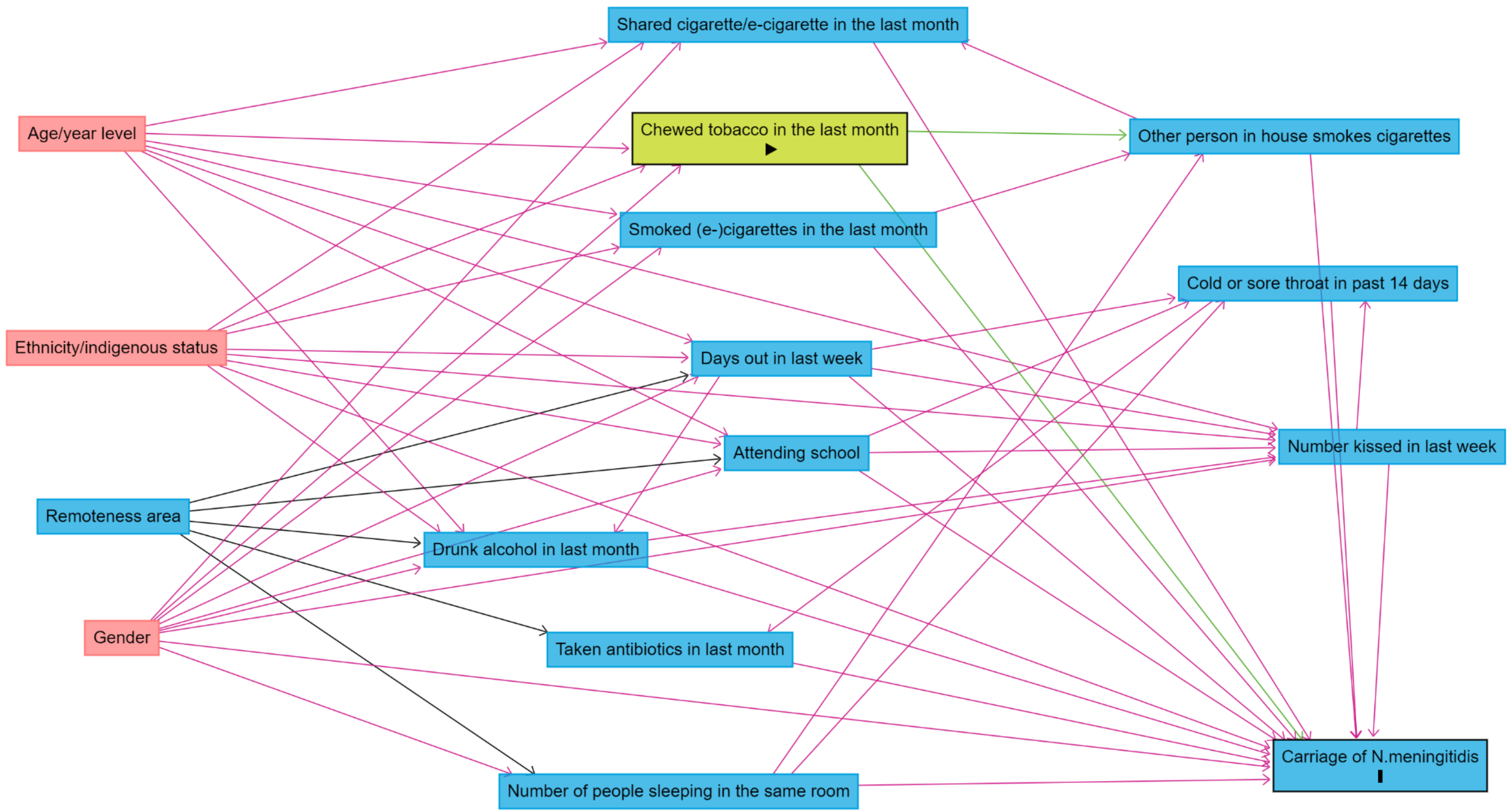

Exposure: Chewed tobacco in the last month

Outcome: Carriage of N.meningitidis

Biasing paths are open.

Minimal sufficient adjustment sets for estimating the total effect of Chewed tobacco in the last month on Carriage of N.meningitidis:

- Age/year level, Ethnicity/indigenous status, Gender

Other person in house smokes cigarettes

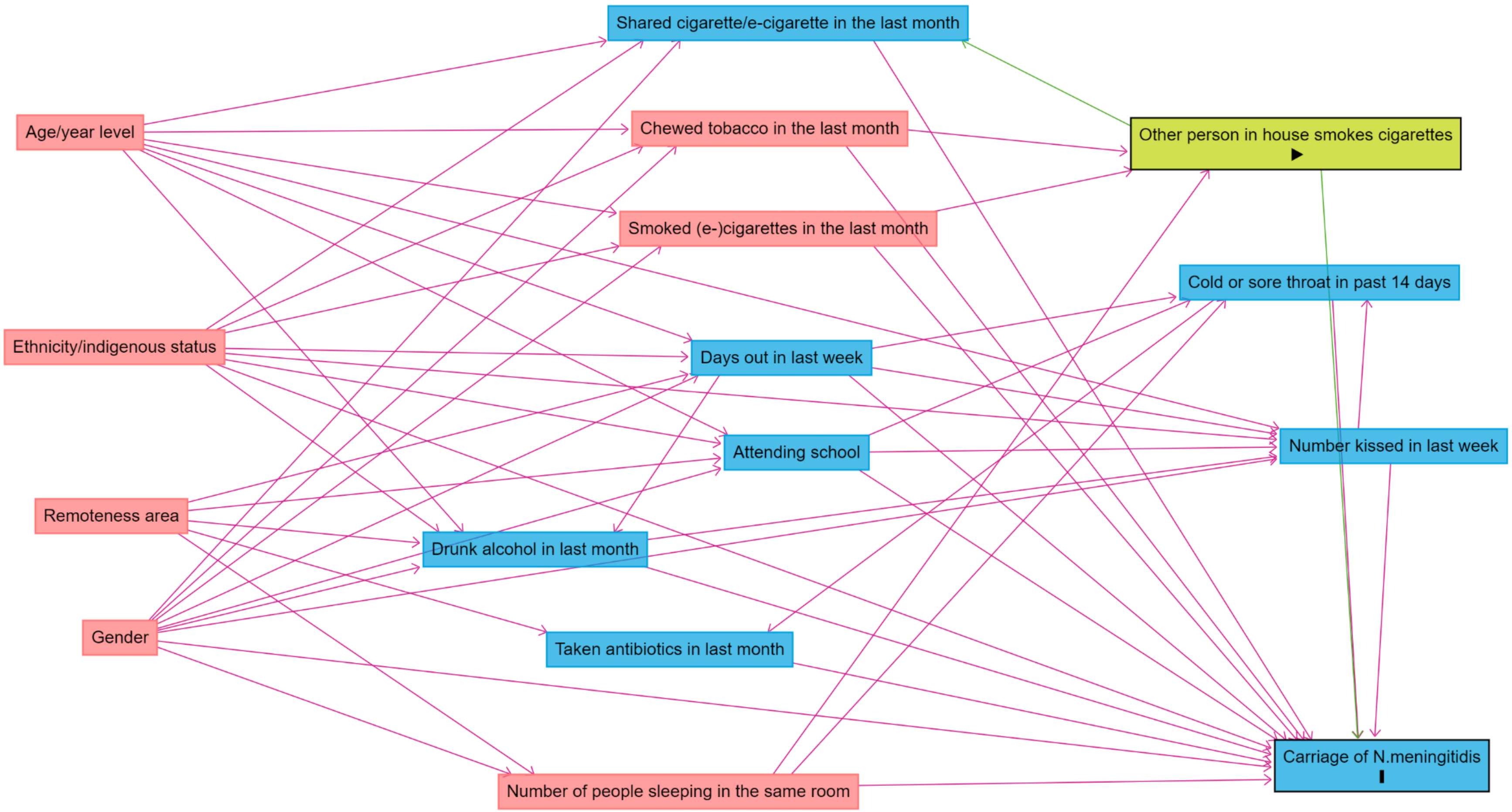

Exposure: Other person in house smokes cigarettes

Outcome: Carriage of N.meningitidis

Biasing paths are open.

Minimal sufficient adjustment sets for estimating the total effect of Other person in house smokes cigarettes on Carriage of N.meningitidis:

- Chewed tobacco in the last month, Number of people sleeping in the same room, Smoked (e-)cigarettes in the last month

Days out in last week (party pub/bar/club/sporting event/gathering/funeral)

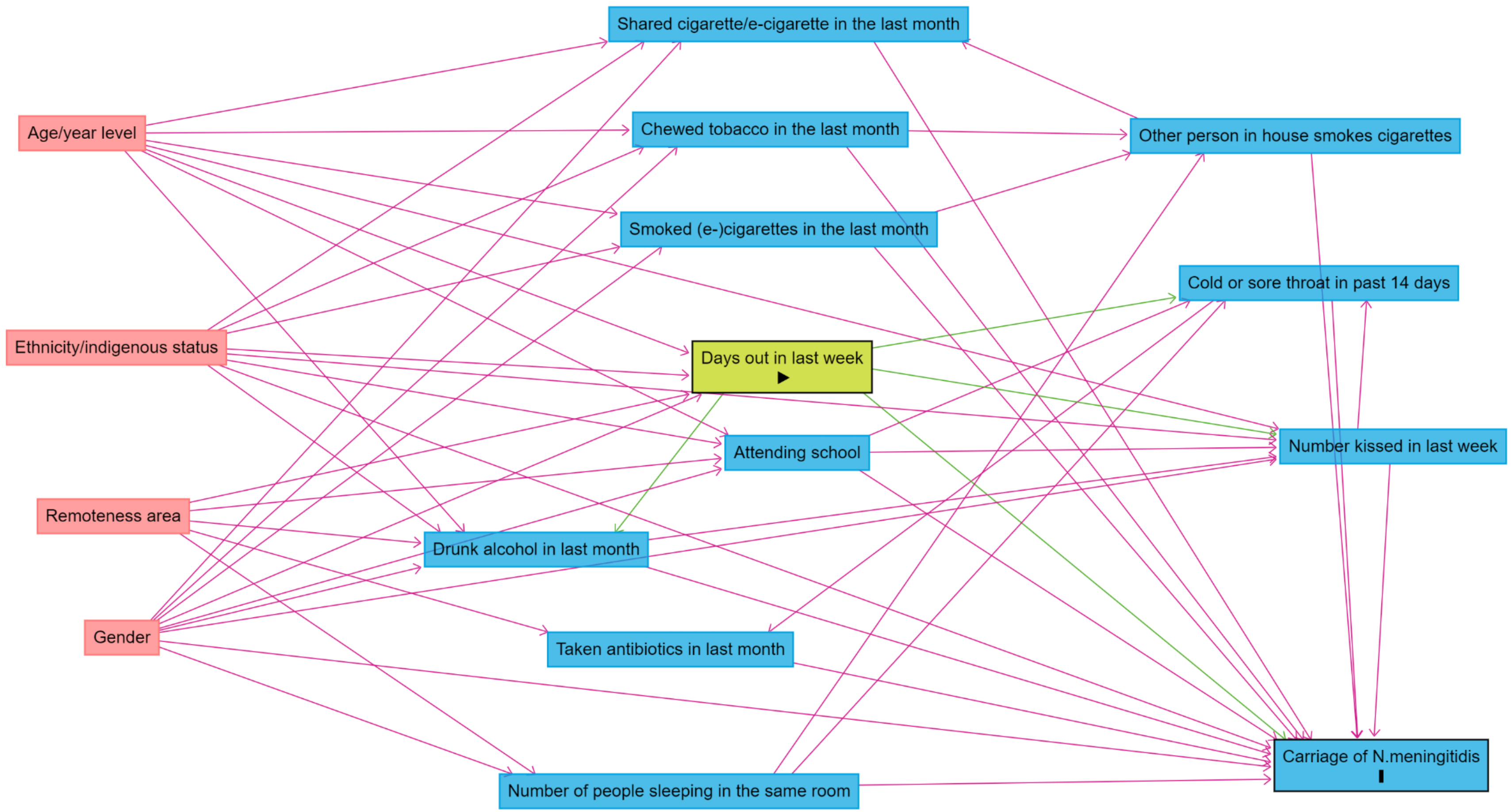

Exposure: Days out in last week

Outcome: Carriage of N.meningitidis

Biasing paths are open.

Minimal sufficient adjustment sets for estimating the total effect of Days out in last week on Carriage of N.meningitidis:

- Age/year level, Ethnicity/indigenous status, Gender, Remoteness area

Drunk alcohol in last month/ No. days in last week drunk alcohol

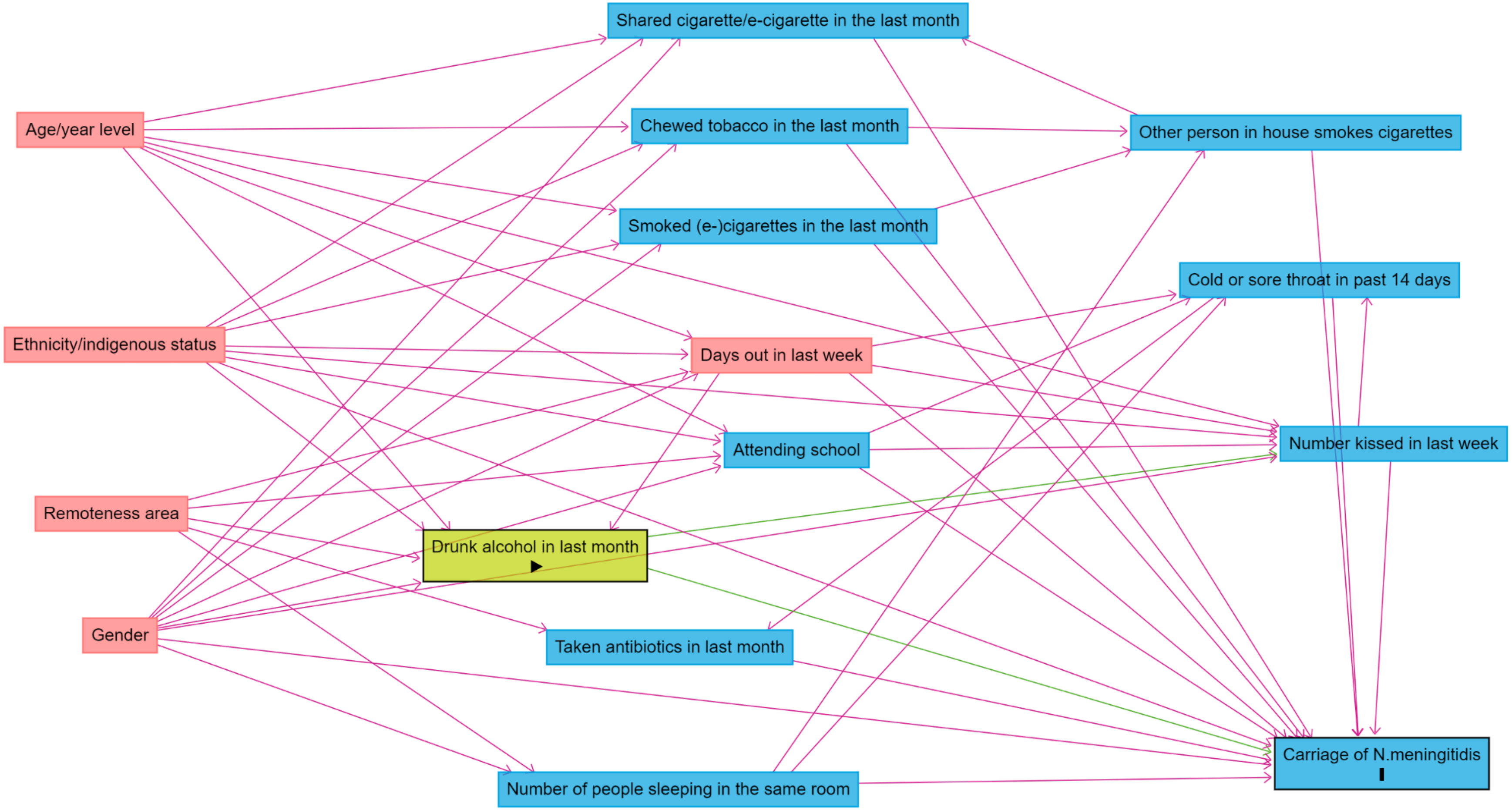

Exposure: Drunk alcohol in last month
Outcome: Carriage of N.meningitidis
Biasing paths are open.
Minimal sufficient adjustment sets for estimating the total effect of Drunk alcohol in last month on Carriage of N.meningitidis:

- Age/year level, Days out in last week, Ethnicity/indigenous status, Gender, Remoteness area

No. people kissed in last week

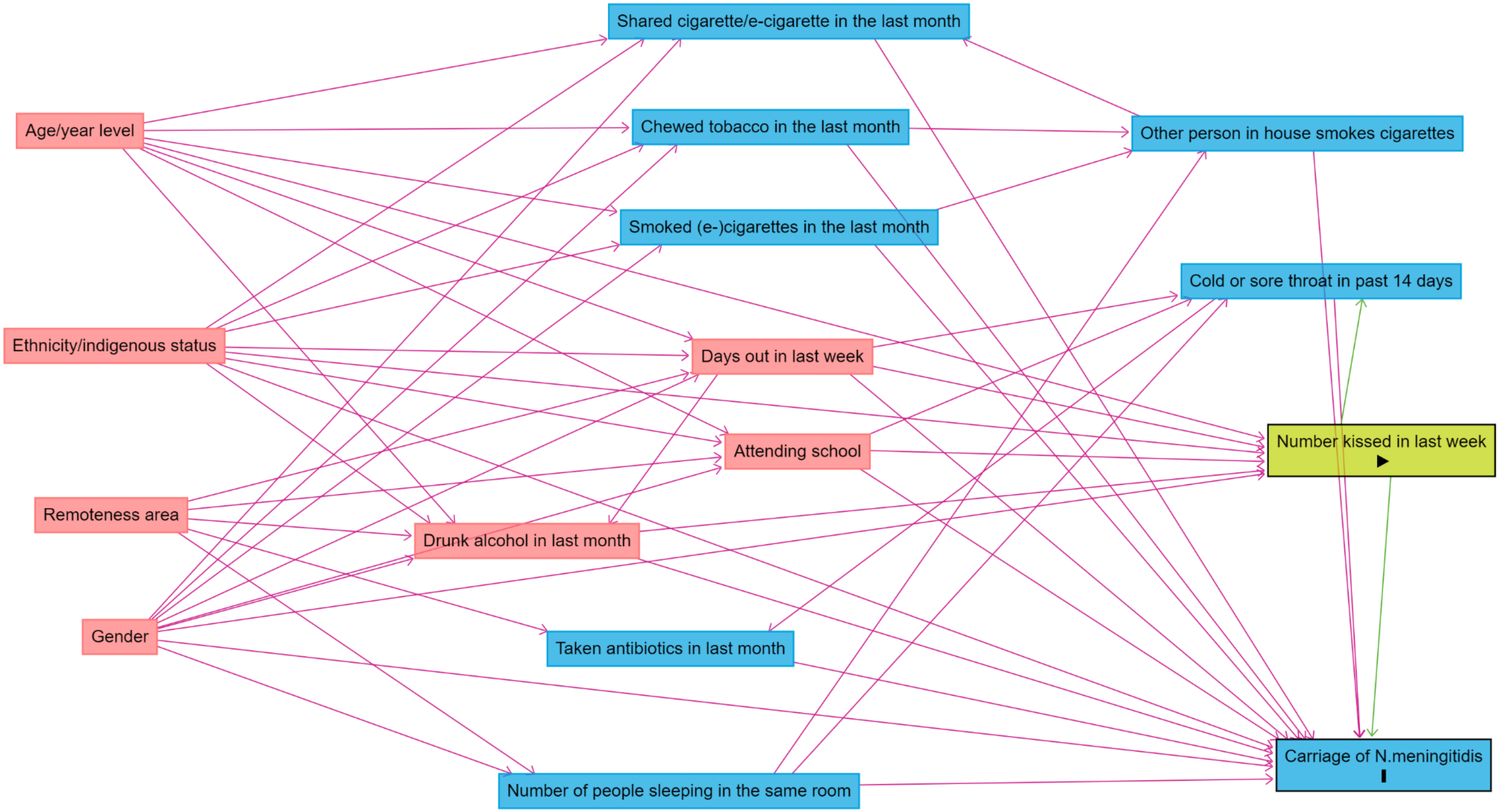

Exposure: Number kissed in last week

Outcome: Carriage of N.meningitidis

Biasing paths are open.

Minimal sufficient adjustment sets for estimating the total effect of Number kissed in last week on Carriage of N.meningitidis:

- Age/year level, Attending school, Days out in last week, Drunk alcohol in last month, Ethnicity/indigenous status, Gender

Number of people sleeping in the same room

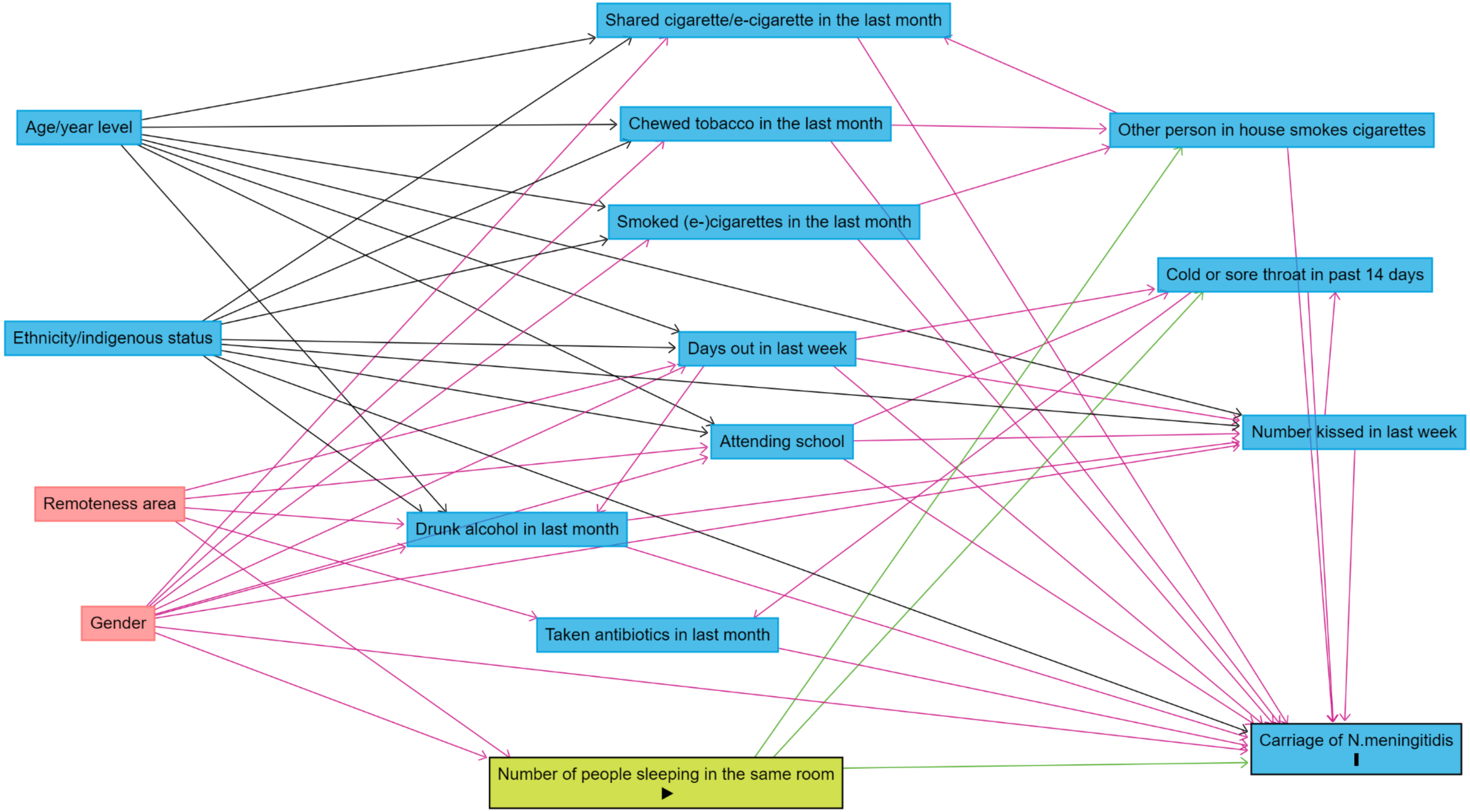

Exposure: Number of people sleeping in the same room
Outcome: Carriage of N.meningitidis
Biasing paths are open.
Minimal sufficient adjustment sets for estimating the total effect of Number of people sleeping in the same room on Carriage of N.meningitidis:

- Gender, Remoteness area
